# Diagnostic Accuracy of Blood-Based Biomarker Panels: A Systematic Review

**DOI:** 10.3389/fnagi.2022.683689

**Published:** 2022-03-11

**Authors:** Anette Hardy-Sosa, Karen León-Arcia, Jorge J. Llibre-Guerra, Jorge Berlanga-Acosta, Saiyet de la C. Baez, Gerardo Guillen-Nieto, Pedro A. Valdes-Sosa

**Affiliations:** ^1^The Clinical Hospital of Chengdu Brain Science Institute, MOE Key Lab for Neuroinformation, University of Electronic Science and Technology of China, Chengdu, China; ^2^Centro de Ingeniería Genética y Biotecnología, La Habana, Cuba; ^3^Centro de Neurociencias de Cuba, La Habana, Cuba; ^4^Instituto de Neurología y Neurocirugía, La Habana, Cuba

**Keywords:** Alzheimer’s disease (AD), blood-based biomarker, diagnosis, biomarker panel, preclinical AD

## Abstract

**Background:**

Because of high prevalence of Alzheimer’s disease (AD) in low- and middle-income countries (LMICs), there is an urgent need for inexpensive and minimally invasive diagnostic tests to detect biomarkers in the earliest and asymptomatic stages of the disease. Blood-based biomarkers are predicted to have the most impact for use as a screening tool and predict the onset of AD, especially in LMICs. Furthermore, it has been suggested that panels of markers may perform better than single protein candidates.

**Methods:**

Medline/Pubmed was searched to identify current relevant studies published from January 2016 to December 2020. We included all full-text articles examining blood-based biomarkers as a set of protein markers or panels to aid in AD’s early diagnosis, prognosis, and characterization.

**Results:**

Seventy-six articles met the inclusion criteria for systematic review. Majority of the studies reported plasma and serum as the main source for biomarker determination in blood. Protein-based biomarker panels were reported to aid in AD diagnosis and prognosis with better accuracy than individual biomarkers. Conventional (amyloid-beta and tau) and neuroinflammatory biomarkers, such as amyloid beta-42, amyloid beta-40, total tau, phosphorylated tau-181, and other tau isoforms, were the most represented. We found the combination of amyloid beta-42/amyloid beta-40 ratio and APOEε4 status to be most represented with high accuracy for predicting amyloid beta-positron emission tomography status.

**Conclusion:**

Assessment of Alzheimer’s disease biomarkers in blood as a non-invasive and cost-effective alternative will potentially contribute to early diagnosis and improvement of therapeutic interventions. Given the heterogeneous nature of AD, combination of markers seems to perform better in the diagnosis and prognosis of the disease than individual biomarkers.

## Introduction

According to the World Alzheimer Report 2019 (Alzheimer’s Disease International, 2019), there are over 50 million people affected by Alzheimer’s disease (AD) worldwide, and it is predicted that the incidence will rise to 152 million by 2050 because of increase in lifespan. AD disproportionately impacts racial and ethnic minority groups and socioeconomically disadvantaged adults. Majority of individuals with dementia (66%) live in low- and middle-income countries, which is estimated to rise to 71–72% by 2050 (Alzheimer’s Disease International, 2019). Significant social and economic burdens fall upon those diagnosed with AD and their families (Sousa et al., 2009; Gilmore-Bykovskyi et al., 2019).

Neuropathological hallmarks of AD include the presence of amyloid plaques, neurofibrillary tangles, and astrogliosis leading to neurodegeneration, as evidenced by neuronal and synaptic loss (McKhann et al., 1984; Serrano-Pozo et al., 2011; Thal et al., 2013; Deture and Dickson, 2019). Various hypotheses have been described regarding AD’s etiology (Davies and Maloney, 1976; Hardy and Higgins, 1992; Mattson et al., 1992; De Knijff and Van Duijn, 1998; Hardy, 2006; Frost et al., 2009; Hardy, 2009; Swerdlow and Khan, 2009; Bagyinszky et al., 2017; Cubinkova et al., 2018; de la Torre, 2018; Hampel et al., 2018; Kimura and Yanagisawa, 2018; Chen and Mobley, 2019; Al Mamun et al., 2020), including the amyloid cascade hypothesis, which claims that cerebral amyloid-β (Aβ) deposition is the pathogenic driving factor of AD, being the most predominant over the last 25 years. However, a common problem of these hypotheses is that they fail to acknowledge other factors and molecular pathways that may mediate the progression of the disease (Banik et al., 2015; Gong et al., 2018). Despite the recent approval of aducanumab, there has been limited progress on therapeutic clinical trials with anti-amyloid agents, which places the amyloid hypothesis as an object of great debate within the scientific community (Ricciarelli and Fedele, 2017; Liu and Yu, 2019; Daly et al., 2020). AD has a multifactorial nature due to coexistence of genetic, epigenetic, biological, and environmental susceptibility. Parallel to the amyloid hypothesis, other causative factors should be investigated and treated simultaneously for better clinical outcomes in AD trials (Banik et al., 2015).

Recent advances in AD research highlight that pathophysiological changes leading to the disease begin decades before the onset of the first symptoms (Aisen et al., 2017). Nevertheless, the heterogeneity of AD clinical manifestations has hurdled the differential diagnosis over other types of dementia, which has been historically made through post-mortem neuropathologic evaluation or, rarely, by brain biopsy in living patients. In 2018, after the redefinition of the disease by the National Institute on Aging and Alzheimer’s Association (NIA-AA), AD has been referred to as an aggregate of neuropathologic changes defined *in vivo* by biomarkers and post-mortem examination (Jack et al., 2018).

**FIGURE 1:**
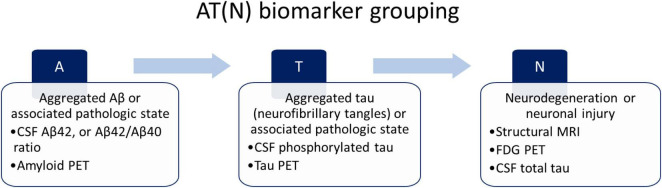
National Institute on Aging and Alzheimer’s Association (NIA-AA) proposed AT(N) biomarker grouping.

Alzheimer’s disease is considered a continuum with three phases: the preclinical stage characterized by normal cognitive ability, the prodromal stage characterized by mild cognitive impairment (MCI), and, ultimately, clinically apparent dementia (Jack et al., 2018; Lilford and Hughes, 2018; Vermunt et al., 2019). The current dementia diagnosis due to AD requires a combination of clinical, neuropsychology, and biomarker measurements. Validated biomarkers used for AD diagnosis are: (i) cerebrospinal fluid (CSF) analysis of Aβ42, t-tau, and p-tau accumulation, and (ii) positron emission tomography (PET) scan (amyloid PET and tau PET). However, even if these methods present high accuracy, the costs and invasiveness do not make them suitable screening tools for the early diagnosis of Alzheimer’s disease. Biomarkers are required to improve diagnosis and monitor the progression of AD (Jellinger et al., 2008). Based on the nature of the pathologic process, biomarkers for AD can be classified into three main groups according to the AT(N) system (Jack et al., 2018): biomarkers of Aβ plaques (A), biomarkers of fibrillar tau (T), and biomarkers of neurodegeneration or neuronal injury (N). Individuals can be categorized into one of the three categories described by the NIA-AA (Figure 1).

Current advances in CSF and PET as biomarker analysis and new identification tools can improve AD diagnostic process. However, these methods have practical limitations that impede their widespread application as screening and first-line diagnostic tools (Table 1). Because of high prevalence of AD in middle and low-income countries, there is an urgent need for inexpensive and minimally invasive diagnostic tests to detect biomarkers in the earliest and asymptomatic stages of the disease. Alternatives such as blood-based biomarkers are predicted to have the most impact for use as a screening tool, and it has been suggested that panels of markers perform better than single protein candidates in terms of sensitivity and specificity for the diagnosis, prognosis, and characterization of AD (Zetterberg and Burnham, 2019). Blood-based biomarkers may represent the first step in the AD’s multistage diagnosis, therefore, this systematic review aims to provide an update on the research and development of AD blood-based biomarkers panels and their diagnostic applications for the prediction of AD, accessible to middle- and low-income countries.

**TABLE 1:** Advantages and disadvantages of current Alzheimer’s disease (AD) diagnostic assessments.

Method	Advantages	Disadvantages
CSF	- High accuracy for diagnosing AD - Relatively cheap in many countries - Enable analyses of markers of inflammation, tau pathology, and neurodegeneration	- Invasive (lumbar puncture)
PET	- High accuracy for diagnosing AD - Can be used in patients that have contraindications for lumbar puncture or when the measure of Aβ 42 in CSF does not fit with the clinical symptomatology	- Expensive - Use radiation - Limited availability

## Methods

### Search Strategy and Eligibility Criteria

A systematic review was completed to identify and describe current blood-derived biomarker panels for AD characterization, diagnosis, and prognosis. A search strategy was developed with the assistance of a research committee formed by dementia experts, clinical researchers, molecular biologists, and bioinformaticians. The research committee provided feedback and guidance on proposed search strategies, selection criteria, and data analysis approach.

The strategy was created using a combination of controlled vocabulary terms and keywords and carried out on the PubMed database for articles published in English or Spanish between January 2016 and December 2020. The following medical subject heading terms were used: “Alzheimer’s disease,” “biomarker,” “panel” (“combined biomarkers” or “signature” or “model”), “blood,” “accuracy” (“AUC” or “ROC” or “specificity” and “sensibility”), “diagnosis,” “amyloid,” “tau,” “neurodegeneration,” and “neuroinflammation.” Articles were included if they contain data on blood biomarkers of neurodegeneration, Aβ or tau pathology, immune response, neuroinflammation, and other biomarker panels for the diagnosis of Alzheimer’s disease. We considered case-control studies, longitudinal studies, clinical trials, and introductory articles addressing pathogenesis and biomarkers for diagnosis of Alzheimer’s disease that were published in peer-reviewed journals. Reviews, abstracts, and editorial letters were excluded. No other filters or limits were applied to the search. To supplement database searches, relevant studies not included in the initial search were recommended by the author’s committee. All citations retrieved by these methods were compiled and screened for appropriateness against the inclusion and exclusion criteria.

**FIGURE 2:**
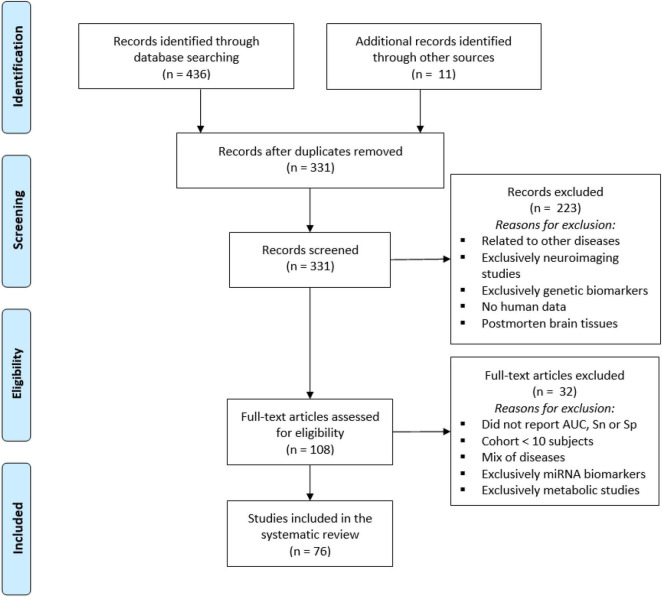
Preferred Reporting Items for Systematic Review and Meta-Analysis (PRISMA) flow diagram for identification of blood-based biomarkers for diagnosis of Alzheimer’s disease detailing the number of abstracts screened and full texts retrieved.

Studies were selected based on preliminary screening of titles and abstracts. A second screening was carried out to identify biomarker panels for the early diagnosis of AD. Articles were excluded if they did not contain an Alzheimer’s disease cohort or a mild cognitive impairment due to Alzheimer’s disease cohort and their controls; had cohorts with less than ten individuals or individuals younger than 18 years of age; had cohorts representing a mix of diagnosis (e.g., Parkinson, Lewy Body, or Vascular dementia); did not report accuracy or efficacy in terms of area under the curve (AUC), sensitivity (Sn), or specificity (Sp) of the biomarkers or marker panels; or had biomarker data from CSF, urine, saliva, neuroimages, genomics studies or cell-based biomarkers. The reference management software Mendeley was used for screening purposes. Finally, studies reporting non-protein biomarkers (e.g., miRNA and metabolites) were excluded for the final selection. The study workflow is described in Figure 2.

### Data Collection

One author (AH-S) extracted data, and two researchers (KL-A and JL-G) checked for accuracy. This study was reported according to the Preferred Reporting Items for Systematic Review and Meta-Analysis (PRISMA) guidelines.

### Synthesis of Results

All studies were summarized in tabular form (Supplementary Table 1). The following information was extracted from the selected articles: name of first author, year of publication, study design, cohort, sample size, blood component studied (tissue), analytical technique for biomarker evaluation, biomarker type (i.e., diagnostic, prognostic, or predictive), proposed biomarker panels for AD diagnosis, and panel performance characteristics (i.e., AUC, Sn, and Sp). Articles were classified based on the blood component studied, and the proposed protein-based markers investigated in panels for AD diagnosis were classified based on AD pathology. Studies on marker panels in plasma with an overlap in more than one biomarker and the panels’ performance characteristics were summarized in tabular form.

## Results

### Study Selection

We identified 436 articles from the initial PubMed search (date range January 2016–December 2020), and 11 were added from other sources. One hundred sixteen duplicated records were removed for a total of 331 unique citations. After a preliminary screening of titles and abstracts, 223 records were excluded because of the following reasons: related to other diseases such as Parkinson’s disease, Down syndrome, cancer, or HIV; articles referring to non-peripheric, neuroimaging, or genetic biomarkers; animal models or studies with post-mortem brain tissues (Figure 2).

We performed a second screening to identify blood-based biomarkers panels for the early diagnosis of AD. Of the 108 full-text articles assessed for eligibility, 32 studies were excluded as they did not report the biomarker’s performance in terms of AUC, Sn, or Sp, had investigated cohorts with less than ten subjects or a mix of diseases, or reported metabolites and genetic biomarkers (Figure 2). Seventy-six articles met the final criteria to be included in the systematic review.

### Study Characteristics

The 76 studies included in the review were classified as case-control (60 studies) or longitudinal (16 studies) with ≥ 2-year follow-up. Most of the studies investigated AD cases vs. healthy controls or conversion from MCI (aMCI, EMCI, LMCI) to AD. The majority of reports (53 studies) were on biomarkers for AD diagnosis, whereas a minority of studies explored prognostic (7 studies) and predictive biomarkers (16 studies). Immunoassays were the preferred analytical technique for evaluating the potential biomarker’s levels in blood. A detailed summary of the study characteristics is available in Supplementary Table 1.

For this review, we focused on potential protein-based biomarkers for AD diagnosis in its earliest stages. The majority of reports studied biomarkers in plasma and serum, whereas a minority explored neuronal-enriched extracellular vesicles, platelets, and red blood cells (Table 2). Conventional biomarkers Aβ and tau, and neuroinflammatory biomarkers were the most represented (Table 3). Measures of amyloid beta-42 (Aβ42), amyloid beta-40 (Aβ40), total tau (T-tau), phosphorylated (p)-tau181, brain-derived neurotrophic factor (BDNF), and complement C3 were proposed as components of panels in three or more studies. Four studies investigated the use of Aβ oligomers as a component of potential biomarker panels for AD early diagnosis.

**TABLE 2:** Number of AD biomarkers and studies for different blood components.

Blood component	Number of biomarkers	Number of studies	Tested parameters and number of studies
Plasma	86	48	Aβ pathology (26), neuroinflammation (8), tau pathology (5), neurodegeneration (3), cholinergic dysfunction (1), oxidative stress (1), energy metabolic dysfunction (1), AD (3)
Serum	41	15	Aβ pathology (6), neuroinflammation (5), tau pathology (1), vascular dysregulation (1), neurodegeneration (1), AD (1)
Exosomes	12	4	Neurodegeneration (2), Aβ and tau pathology (1), tau pathology (1)
Platelets	4	3	Aβ pathology (2), tau pathology (1)
NeV	8	3	Tau pathology (1), neuroinflammation (1), AD (1)
RBC	2	1	α-synuclein pathology

*NeV, Neuronal-enriched extracellular vesicles; RBC, red blood cells.*

**TABLE 3:** Candidate blood-based marker classification according to AD pathology.

Category	Biomarker
Aβ pathology	Aβ40, Aβ42,Aβ42/Aβ40, APP/Aβ42, Aβ oligomers, Aβ secondary structure, Aβ misfolding, APP, A2M, ACE, NCAM, AHI1, APLP2, GSN, SAP, TTR *APP metabolism:* BACE1, ADAM10, PSEN1, cathepsin D
Tau markers	Tau, T-tau, T-tau/Aβ42, p-tau181, Alz-tau^®^, pSer312-IRS-1, pY-IRS-1
Neuroinflammation	IgM, IgM-1, VCAM-1, A1M, AHSG, PPC1I, TIMP1,MMP-1, MMP-3, MMP-9, CRP, sFLT-1, sICAM-1, Tie-2, CD200, sCD40L, EOT1,EOT3, FB, FH, sCR1, LGALS3BP, OPN, TNC, CLEC1B, A1AT, B2M, FCN2 *Cytokines:* IL-1α, IL-3, IL-8, IL-10, IL-12/23p40, IL-13, IL-16, IL-17, IL-18, TNFα, OSM, IFN-α2 *Chemokines:* CXCL10, CXCL13, CCL11, CCL20, MCP-1 *Complement:* C3, aC3/nC3, C4, CFI *Growth factors:* TGF-β1, VEGF, VEGF-C, VEGF-D, bFGF, IGF-1, IGFBP-2
Neurodegeneration	CgA, BDNF, GFAP *Neuronal injury:* *NF-L* *Synaptic dysfunction:* Ng, SNAP-25, SYT, GAP43, VLDLR, SYNPO, NRGN, SYP
Lipid metabolism	APOE, apoA-I
Oxidative stress	ApoJ, Klotho, protein carbonyls, circulating-proteosome
Vascular dysregulation	FAC, FBC, uPA
Energy metabolic dysfunction	AMPKα1
α–synuclein pathology	α-syn/Aβ, α-syn/tau
Cholinergic dysfunction	AChE
Other	PPY, DYRK1A, AAT, ACT, KLK8, AGT, AXIN1, CDH5, HAGH, HCY, POSTN, PPP, ECH1, HOXB7, NHLRC2, FN1, ERBB2, SLC6A13, unfolded p53

### Studies Assessing Biomarker Panels

Several blood-based biomarker panels have proven high sensitivity and specificity to predict CSF Ab/tau levels and amyloid and tau burden as measure by PET. After comparing the most recent panels, we found the ratio Aβ42/Aβ40 combined with APOEε4 status in plasma to be the most represented (6 studies) with high accuracy for predicting Aβ-PET status (Table 4). Furthermore, three articles proposed the marker Aβ42 as a component of biomarker panels in plasma for AD prediction with high accuracy using CSF Aβ42, T-tau, and p-tau181 as reference standards. Moreover, according to two studies, the combination of Aβ42, CgA, and EOT3 with APOEε4 status seems to provide a high predictive value (AUC = 0.84) for CSF Aβ42 status.

**TABLE 4:** Blood-derived biomarker panels for prediction of cerebrospinal fluid (CSF) and PET status as gold standards.

Overlapped biomarkers								Panel	AUC	Reference standard	Subjects (*N*) and disease stage	Subjects characteristics (*n*, mean ± SD)	References
Aβ42	Aβ42/Aβ40	APOEε4	APOE	CgA	EOT3	NF-L	pTau181						
	■	■						Aβ42/Aβ40, APOEε4 status	0.88–0.913*^a^*	Aβ-PET	*N* = 176 HC, MCI, AD	Month 18 *n* = 176, avg. age: 73.7 ± 7.2, f/m: 86/90 Month 36 *n* = 169, avg. age: 75 ± 7.1, f/m: 83/86 Month 54 *n* = 135, avg. age: 76.9 ± 7.1, f/m: 65/70	Doecke et al., 2020
	■	■						Aβ42/Aβ40, APOEε4 status	0.83 (95% CI 0.77–0.89; Sn = 76%; Sp = 75%)*^a^*	CSF Aβ,Aβ-PET	*N* = 248 SCD	*N* = 248, avg. age: 61 ± 9, f/m: 103/145	Verberk et al., 2018
	■	■						Aβ42/Aβ40, APOEε4 status	0.78	Aβ-PET	*N* = 95 SMC, non-SMC	Aβ− *n* = 63, avg. age: 77.65 ± 5.62, f/m: 44/19 Aβ+ *n* = 32, avg. age: 79.50 ± 5.32, f/m: 19/13	Chatterjee et al., 2019
	■	■						Aβ42/Aβ40, APOEε4 status	0.519 (APOEε4+) 0.648 (APOEε4−)	Aβ-PET	*N* = 117 APOEε4+, APOEε4−	APOEε4+ *n* = 28, avg. age: 71.6 ± 11.2, f/m: 18/10 APOEε4− *n* = 89, avg. age: 71.7 ± 12.2, f/m: 50/39	Tateno et al., 2017
	■	■				■		Aβ42/Aβ40, APOEε4 status, tau, NF-L	Cohort 1: 0.80–0.87; Cohort 2: 0.86	CSF Aβ42/Aβ40	*N* = 1079 CU, MCI, AD	Cohort 1 *N* = 842, avg. age: 72 ± 5.6, f/m: 446/396 Cohort 2 *n* = 237, avg. age: 66 ± 10, f/m: 120/117	Palmqvist et al., 2019
	■					■		Aβ42/Aβ40, GFAP, NF-L	Total: 0.88 (Sn = 0.82, Sp = 0.86) Non-demented: 0.84 (Sn = 0.70, Sp = 0.86)	Aβ-PET	*N* = 252 SCD, MCI, AD	PET+ *n* = 176, avg. age: 63 ± 7 years, f/m: 87/89 PET− *n* = 76, avg. age: 61 ± 9 years, f/m: 27/49	Verberk et al., 2020
	■						■	Aβ42/Aβ40, pTau181	0.84 (95% CI = 0.79–0.89)	Amyloid PET, Tau PET, CSF P-tau181	Cohort 1: *N* = 182 Cohort 2: *N* = 344 Preclinical AD, MCI, AD	Cohort 1 *N* = 182, avg. age: 72, f/m: 79/103 Cohort 2 *n* = 344, avg. age: 71, f/m: 174/170	Janelidze et al., 2020
**■**		■		■	■			Aβ42, CgA, EOT3, APOEε4 status	0.84 (Sn = 0.82; Sp = 0.62; PPV = 0.81; NPV = 0.64)	CSF Aβ42	*N* = 358 HC, MCI, AD	HC *n* = 58, avg. age: 75.11 ± 0.77, f/m: 28/30 MCI *n* = 198, avg. age: 74.37 ± 7.49, f/m: 65/133 AD *n* = 102, avg. age: 74.86 ± 7.88, f/m: 43/59	Eke et al., 2020
**■**		■	■	■	■			Aβ42, APOE, CgA, EOT3, APOEε4 status	0.84 (Sn = 0.78, Sp = 0.73)	CSF Aβ42, tTau, and pTau181	*N* = 566 HC, MCI, AD	Validation: MCI *n* = 198, avg. age: 75.13 ± 7.32, f/m: 75/123 AD *n* = 10, avg. age: 73.73 ± 10.04, f/m: 4/6	Goudey et al., 2017
**■**							■	Brain derive dexosomal Aβ42, pTau181, T-tau	discovery cohort: 0.86–0.97; validation cohort: 0.85–0.98	CFS Aβ42, T-tau, and P-T181-tau	*N* = 298 HC, aMCI, AD	*N* = 298, avg. age: 65 ± 6, f/m: 162/136	Jia et al., 2019

*Aβ42, amyloid-β42; Aβ42/Aβ40, amyloid-β 42-40 ratio; APOE, apolipoprotein; T-tau, total tau; pTau181, phosphorylated tau 181; CgA, chromogranin-A; EOT3, eotaxin 3; AUC, area under the curve; CI, confidence interval; CSF, cerebrospinal fluid; AD, Alzheimer’s disease; aMCI, amnestic mild cognitive impairment; SCD, subjective cognitive decline.*

*^a^Age, gender, and presence of APOEε4 allele were included as covariates. The black squares show the overlapping biomarkers from the different studies.*

Other articles proposed the use of biomarker panels for AD prognosis and characterization. Among all the potential markers proposed, Aβ42, which has a significant role in Aβ pathology, brain-derived neurotrophic factor (BDNF), which may have a role in cognitive dysfunctions in AD, and complement C3, which has been reported as a marker of brain atrophy in AD and amyloidosis in non-demented elderly, were the most repeated in the panels (Table 5). Complement C3 in combination with A1-microglobulin (A1M), A2-macroglobulin (A2M) reported high accuracy in panels for discriminating between AD and healthy individuals, whereas Aβ42 and BDNF were found in combination with various biomarkers in the studies. In addition, the combination of cytokine interleukin 13 (IL-13) and C-X-C motif chemokine ligand 10 (CXCL10) showed high diagnostic values in two separate studies.

**TABLE 5:** Multivariate panels with overlapping biomarkers.

Overlapped biomarkers													Panel	Statistics	Subjects (*N*) and disease stage	Subjects characteristics (*n*, mean ± SD)	References
Aβ42	A1M	A2M	BDNF	C3	C4	Cathepsin D	CXCL10	IL13	IL10	TNFα	TTR	VEGF					
	■	■		■									A1M, A2M, C3, IgM, TNC	AUC = 0.89; Sn = 86.5%; Sp = 82.1%; Accuracy = 85%	*N* = 166 HC, AD	AD *n* = 108, avg. age: 74.6 ± 8, 42.6% female HC *n* = 58, avg. age: 75.1 ± 5.8, 48.3% female	Eke et al., 2018
	■	■		■									A1M, A2M, C3, AAT, APOE, PPP	Sn = 85.4%, Sp = 78.6%	*N* = 157 HC, AD	AD *n* = 106, avg. age: 74.88 HC *n* = 51, avg. age: 74.56	Jammeh et al., 2016
				■							■		APOA, C3, TTR	AUC = 0.89; Sn = 83%; Sp = 90%	*N* = 63 HC, EMCI, LMCI, AD	HC *n* = 10, avg. age: 62.6 ± 8.3, f/m: 8/2 EMCI *n* = 26, avg. age: 65.5 ± 10.5, f/m: 19/7 LMCI *n* = 23, avg. age: 69.9 ± 9.6, f/m: 9/14 AD *n* = 4, avg. age: 77.0 ± 3.7, f/m: 3/1	Liu et al., 2019
							■	■					IL-13,CXCL10	AUC = 1 (95% CI); Sp = 100%; Sn = 100%	*N* = 78 HC, AD	AD *n* = 39, avg. age: 80.7 ± 6.41, f/m: 22/17 HC *n* = 39, avg. age: 72.1 ± 5.04, f/m: 15/24	Mohd Hasni et al., 2017
							■	■		■			IL-13, IL-1α, CXCL10, IL-3, TNFα	AUC = 0.99 (Sp = 88.6%; Sn = 97.7%; *p* < 0.05)	*N* = 312 HC, AD	AD *n* = 156, avg. age: 72.69 ± 10.15, f/m: 81/75 HC *n* = 156, avg. age: 74.40 ± 9.15, f/m: 63/93	Yu et al., 2017
					■	■							BDNF, AGT, IGFBP-2, OPN, cathepsin D, SAP, C4, TTR	AUC = 0.958, Sn = 86.7%, Sp = 88.1%, Accuracy = 87.4%	*N* = 199 HC, AD	AD *n* = 98, avg. age: 78.76 ± 8.06, f/m: 64/34 HC *n* = 101, avg. age: 78.33 ± 7.30, f/m: 66/35	Cheng et al., 2018
					■	■							FCN2, CFI, C4, B2M, Cathepsin D, APOEε4, A1AT	AUC = 0.742, Sn = 0.682, Sp = 0.704	*N* = 1866 HC, MCI, AD	Cohort 1: *n* = 457 (normal Aβ), avg. age: 66.52 ± 8.71, f/m: 223/234 *n* = 543 (abnormal Aβ), avg. age: 69.81 ± 8.12, f/m: 301/242 Cohort 2: *n* = 460 (normal Aβ), avg. age: 66.61 ± 8.25, f/m: 253/207 *n* = 406 (abnormal Aβ), avg. age: 70.47 ± 8.22, f/m: 223/180	Westwood et al., 2020
**■**													APP, NCAM, Aβ40, Aβ42	AUC = 0.997, Sn = 98.5	*N* = 126 HC, AD	AD *n* = 96, avg. age: 82.31 ± 5.86, 45.83% male HC *n* = 30, avg. age: 82.03 ± 4.17, 33.33% male	Chen et al., 2019
**■**													Aβ42, APP/Aβ42, Aβ42/Aβ40	Discovery: AUC = 0.967 Validation: AUC = 0.941 Accuracy = 90%	NCGG cohort = 121, AIBL cohort = 252 HC, MCI, AD	Age: 60–90 years, native Japanese	Nakamura et al., 2018
**■**									■	■			Aβ40, Aβ42, MMP-1, MMP-3, IL-8, IL-10, and TNFα	pAD: AUC = 0.732 (95%CI 0.614–0.849). MOCA decline: AUC = 0.751 (95%CI. 0.544–0.958); CAMCOG decline: AUC = 0.844, 95%CI 0.751–0.936	*N* = 107 HC, MCI, SMI, pAD	*N* = 107, avg. age: 78.4 ± 7.2, f/m: 54/53	Iulita et al., 2019
										■			IFNα-2, IL-1, TNFα	AUC = 0.6524	*N* = 289 HC, MCI, AD	HC *n* = 87, avg. age: 75.9 ± 9.0, f/m: 52/35 MCI *n* = 73, avg. age: 77.5 ± 6.3, f/m: 45/28 AD *n* = 129, avg. age: 81.0 ± 6.2, f/m: 92/37	Boccardi et al., 2019
**■**													Aβ42, CXCL13, IgM-1, IL-17, PPY, VCAM-1	Sn = 80%; Sp = 82% Validation cohort: Sn = 79%; Sp = 76%	*N* = 585 HC, MCI, AD	*N* = 585, avg. age: 70.1 ± 6.8, f/m: 349/236	Burnham et al., 2014, 2016
									■				IL-10, IL-12/23p40	18 months: AUC = 0.802 54 months: AUC = 0.805	*N* = 665 HC, MCI, AD	*n* at 18 months/54 months: 559/528 (HC), 39/51 (MCI), 67/86 (AD) Avg. age: 69 ± 6 (HC), 75 ± 6 (MCI), 76 ± 7 (AD) F/M: 325/234 (HC), 17/22 (MCI), 44/23 (AD)	Pedrini et al., 2017
			■								■		BDNF, AGT, IGFBP-2, OPN, cathepsin D, SAP, C4, TTR	AUC = 0.958 (95% CI, 0.934–0.982); Sn = 0.867; Sp = 0.881; Accuracy = 87.4%	*N* = 199 HC, AD	HC *n* = 101, avg. age: 78.33 ± 7.30, f/m: 66/35 AD *n* = 98, avg. age: 78.76 ± 8.06, f/m: 64/34	Cheng et al., 2018
			■									■	BDNF, IGF-1, VEGF, TGF-β1, MCP-1, IL-18	AUC = 0.94; Sn = 76%; Sp = 95%; Accuracy = 85%	*N* = 160 HC, AD	HC *n* = 79, avg. age: 64.5 ± 2.7, f/m: 28/51 AD *n* = 81, avg. age: 81.9 ± 7.8, f/m: 54/27	Schipke et al., 2019
			■										DYRK1A, BDNF, HCY	AUC = 93.3%; Sn = 0.952; Sp = 0.889	*N* = 140 HC, AD	Cohort M *n* = 20 (HC), 69 (AD) Avg. age: 65.2 ± 8.4 (HC), 68.5 ± 7.47 F/M: 7/13 (HC), 35/35 Cohort P *n* = 25 (HC), 26 (AD) Avg. age: 67.9 ± 8.5 (HC), 64.1 ± 8.15 F/M: 12/13 (HC), 13/13	Janel et al., 2017
			■										HCY, BDNF, APOEε4	Sn = 85.0%, Sp = 86.0%, PPV = 0.93, NPV = 0.73%	*N* = 254 HC, MCI-AD, MCI-MCI	HC *n* = 90, avg. age: 76.4 ± 4.4, f/m: 41/49 MCI-AD *n* = 76, avg. age: 68.3 ± 4.2, f/m: 33/43 MCI-MCI *n* = 88, avg. age: 72.6 ± 4.6, f/m: 42/46	Zheng et al., 2016
												■	bFGF, CRP, IL-16, sFLT-1, sICAM-1, Tie-2, VEGF-C, VEGF-D	AUC = 0.89 (0.81–0.95)	*N* = 120 MCI and mild dementia	CDR = 0 *n* = 48, avg. age: 66 ± 7.4, f/m: 31/17 CDR > 0 *n* = 72, avg. age: 73.3 ± 6.9, f/m: 46/26	Popp et al., 2017
												■	VEGF, sCD40L	AUC = 0.58 (95% CI: 0.775–0.941)	*N* = 90 HC, AD	AD *n* = 50, avg. age: 76.42 ± 8.88, f/m: 20/30 HC *n* = 40, avg. age: 77 ± 7.46, f/m: 7/13	Yu et al., 2016

*Aβ, amyloid-β; A1M, A1-microglobulin; A2M, A2-macroglobulin; BDNF, brain-derived neurotrophic factor; C3, complement C3; CXCL, C-X-C motif chemokine ligand; IL, interleukin; TNFα, tumor necrosis factor alpha; TTR, transthyretin; VEGF, vascular endothelial growth factor; IgM, immunoglobulin M; TNC, tenascin C; AAT, alpha-1 antitrypsin; APOE, apolipoprotein E; PPP, pancreatic polypeptide; APP, amyloid precursor protein; NCAM, neural cell adhesion molecule; MMP, matrix metalloproteinase; PPY, pancreatic polypeptide; VCAM-1, vascular cell adhesion protein; AGT, angiotensinogen; IGFBP-2, insulin-like growth factor binding protein 2; OPN, osteopontin; SAP, serum amyloid P component; C4, complement C4; IGF-1, insulin-like growth factor 1; TGF-β1, transforming growth factor beta 1; MCP-1, monocyte chemoattractant protein-1; DYRK1A, dual-specificity tyrosine-(Y)-phosphorylation-regulated kinase 1A; HCY, homocysteine; bFGF, basic fibroblast growth factor; CRP, C-reactive protein; sFLT-1, soluble fms-like tyrosine kinase-1; sICAM-1, soluble intercellular adhesion molecule-1; Tie-2, tyrosine kinase receptor TIE-2; VEGF, vascular endothelial growth factor; sCD40L, soluble CD40 ligand; AUC, area under the curve; CI, confidence interval; CSF, cerebrospinal fluid; AD, Alzheimer’s disease; MCI, mild cognitive impairment; SMI, severe mental impairment; pAD, probably AD. The black squares show the overlapping biomarkers from the different studies.*

## Discussion

Over the past few years, different studies have demonstrated that central nervous system (CNS) disease-associated protein alterations could be detected in blood. Blood testing is a less invasive and cost-effective method that could counteract the limited accessibility and availability of PET due to CSF testing’s expenses and invasiveness. Furthermore, blood testing and blood sample-handling infrastructures are already well established globally for clinical routines and sample collection and processing. Therefore, blood-based biomarker tools can meet the scalability requirements for broad population-based screening and offer the opportunity to measure a wide range of potential pathophysiological biomarkers involved in multifactorial AD molecular mechanisms beyond conventional Aβ and tau pathologies.

**FIGURE 3:**
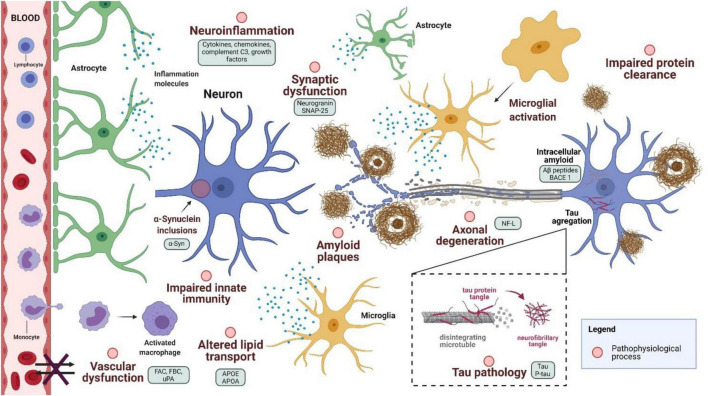
Pathophysiological process in Alzheimer’s disease and proposed biomarkers. SNAP-25, synaptosomal-associated protein, 25 kDa; FAC, fibrinogen α chain; FBC, fibrinogen β chain; uPA, urokinase-type plasminogen activator; APO, apolipoprotein; neurofilament light (NF-L); BACE1, beta-secretase 1 (Created with BioRender.com).

Proteins related to various pathophysiological processes in AD progression have been proposed as new blood-based biomarkers (Figure 3). However, a critical limitation of measuring biomarkers for brain diseases in the blood is the relatively low concentration of these markers due to the permeability of the blood-brain barrier, which prevents the free passage of molecules from the CNS to the blood (Zetterberg and Burnham, 2019). This limitation might require sensitive and specific assays, followed by careful validation studies.

### Plasma Aβ

Plasma Aβ has been reported as a potential predictor of AD; however, results are inconsistent (Winston et al., 2018; Feinkohl et al., 2020). Aβ42 is highly labile and prone to aggregate, making its concentrations susceptible to variation in pre-analytical processing. The ratio of Aβ42/Aβ40 in plasma may be more useful than Aβ peptides individually, and it appears to be associated with an increased risk of progression to AD dementia and more significant cognitive decline. Correlation or partial correlation between Aβ42/Aβ40 plasma ratio and amyloid PET and CSF has been reported (de Rojas et al., 2018; Nabers et al., 2018; Li et al., 2019; Nabers et al., 2019; Pérez-Grijalba et al., 2019), which supports its potential use as a screening tool to identify brain Aβ accumulation in the preclinical and prodromal stages of AD (Pérez-Grijalba et al., 2019).

Nakamura et al. (2018), in a recent study, described a decrease in plasma Aβ42/Aβ40 ratio measured by an immunoprecipitation and liquid chromatography-mass spectrometry assay (IP-MS), providing evidence that plasma Aβ42/Aβ40 can accurately diagnose brain amyloidosis (AUC = 0.88) (Nakamura et al., 2018). This result supports the use of Aβ42/Aβ40 ratio in plasma as a screening tool for those at risk of AD dementia. Lower plasma Aβ42/40 ratio has been associated with a twofold increased risk of clinical progression to MCI or dementia (Verberk et al., 2018). Additionally, it was found that individuals with positive plasma Aβ42/Aβ40 but negative amyloid PET scan have a 15-fold higher risk of converting to amyloid PET-positive (*p* = 0.01), which suggests that Aβ42/Aβ40 ratio becomes positive earlier than the established amyloid PET threshold used in this study. Therefore, positive plasma Aβ42/Aβ40 with negative amyloid PET scan may represent early amyloidosis rather than false positive results in some individuals. Finally, the study revealed that plasma and CSF Aβ42/Aβ40 declined within individuals over time, likely reflecting brain amyloid accumulation in some participants (Nakamura et al., 2018).

Quantifying Aβ peptide levels in the blood by conventional enzyme-linked immunosorbent assay (ELISA) is undoubtedly a demanding laboratory task; it requires considerable expertise because of various technical reasons that compromise obtaining reliable and reproducible results. Assay conditions and the procedure’s complexity are mostly related to the relatively low levels of these peptides in the blood and their high hydrophobic nature. Because of low levels of Aβ peptides in the blood, it is critical to strictly use antibodies with high affinity and strictly standardize the Aβ blood test methodology. In a previous study, Pesini et al. (2012) showed that if sufficient attention is devoted to these issues, the ELISA sandwich colorimetric test is sensitive enough to detect low concentrations of Aβ (LLD < 6 pg/ml) with good intra-assay repeatability and inter-laboratory reproducibility, even with a relatively low number of samples.

Early case-control studies on plasma Aβ42 and Aβ40 using ELISA found no or only minor differences between AD and control groups. Heterogeneity of sample population, small sample size, confounding factors (in particular age), and insufficient analytical sensitivity for the ELISA method are all thought to contribute to the reported result’s low reproducibility (Janelidze et al., 2016). In contrast, plasma Aβ peptides, when measured by IP-MS (Schindler et al., 2019), and an ultrasensitive single molecule array (Simoa) assay (Verberk et al., 2018; Chatterjee et al., 2019) have shown more promising results for detection of Aβ peptides in blood plasma. A nanosheet-based sensor system has recently been studied to quantify Aβ42 and proposed as a non-invasive and cost-effective alternative for early detection of AD (Gao et al., 2020).

Aβ oligomerization has also been subject for study as a potential candidate biomarker for diagnosis of AD (An et al., 2017; Meng et al., 2019; Youn et al., 2020), and it has been reported to be correlated with CSF Aβ42, Pittsburgh compound B (PiB) PET (PiB-PET), CSF phosphorylated tau, and CSF total tau (Wang et al., 2017). In a recent case-control study, Meng et al. (2019) found higher oligomeric Aβ levels in AD plasma than in a control group’s plasma. Correspondingly, oligomeric Aβ concentration measures were distinguished between the two groups with high accuracy (AUC = 0.89; Sp = 90%, and Sn = 82.1%). Besides, elevated levels of Aβ oligomers were correlated with cognitive performance in patients with AD, suggesting that oligomeric Aβ in plasma could be a potential biomarker for AD diagnosis (Meng et al., 2019).

### Plasma Tau

Tau deposition in the brain is one of the hallmarks of AD. Increased levels of CSF t-tau and p-tau protein are well-established biomarkers for AD diagnosis. However, studies have shown inconsistent results in the association of plasma t-tau and p-tau with AD, which might be due to low concentrations of the protein, the heterogeneity of extracellular tau primary structure, and difference between measurement assays used among studies. To address these problems, Chen et al. (2019) developed a set of validated immunoassays to detect tau fragments in CSF and blood plasma. Measurements using an N-terminal assay showed elevated concentration of plasma tau in patients with AD and MCI, and reported high accuracy for discriminating MCI, AD, and the control group [discovery cohort: AUC (MCI) = 0.88, AUC (AD) = 0.96; validation cohort: AUC (MCI) = 0.79, AUC (AD) = 0.75] (Chen et al., 2019). Mielke et al. (2018) demonstrated that plasma p-tau181 and total tau were differentially associated with neuroimaging measures of AD pathology. Both plasma tau and p-tau181 levels were elevated in patients with AD dementia compared to cognitively unimpaired patients. Plasma p-tau181 was a better predictor of elevated Aβ PET than total tau (*P* < 0.01), age (*P* < 0.05), or APOE (*P* < 0.05) alone (AUC = 0.7–0.85), highlighting the potential use of plasma p-tau181 as a non-invasive blood-based screener of AD pathophysiology (Mielke et al., 2018).

Recent findings support the potential use of p-tau217 and p-tau181 as AD plasma biomarkers because of their higher specificity to monitor CNS changes in AD plasma than t-tau and p-tau202 (Barthélemy et al., 2020; Janelidze et al., 2020). Assays have been developed to detect blood tau p-tau181 (Mielke et al., 2018; Karikari et al., 2020), and plasma levels of p-tau181 have been reported to be significantly increased in AD, particularly in symptomatic stages, detecting AD with good accuracy (Mielke et al., 2018; Janelidze et al., 2020; Karikari et al., 2020). Karikari et al. (2020) reported probably the first simple, practical, and scalable test for measuring p-tau181 in plasma and serum. Using Simoa^®^ Technology, Karikari et al. (2020) developed a blood-based immunoassay that showed good diagnostic performance for AD in four different cohorts and appeared to be specific to AD, discriminating it from other neurodegenerative diseases with high accuracy (AUC = 0.82–0.92). Furthermore, plasma p-tau181 identified AD across the clinical *continuum* and performed better than age, APOEε4 genotype or both, and other plasma biomarkers (t-tau, Aβ42, Aβ42/Aβ40 ratio, and t-tau/Aβ42 ratio) in predicting AD, increased tau PET, and increased Aβ PET (Karikari et al., 2020). In a more recent study (Karikari et al., 2021), the performance of plasma p-tau181 was demonstrated in a multi-center study with more than 1000 participants. Increased levels of plasma p-tau181 in the preclinical stage of AD was confirmed. In addition, p-tau181 identified AD dementia with high diagnostic accuracy (AUC = 85.3%; 95% CI 81.4–89.2%), as well as distinguished between Aβ− and Aβ+ individuals along the Alzheimer’s *continuum* (AUC = 76.9%; 95% CI 74–79.8%). These results validate the use of plasma p-tau181 as a promising and accurate diagnostic and prognostic biomarker for AD (Karikari et al., 2021).

Janelidze et al. (2020) also found a correlation between p-tau181 and CSF p-tau181, tau PET, different AD stages. Plasma p-tau181 increases early in the disease around the time point of Aβ positivity, supporting the use of plasma p-tau181 as a noninvasive diagnostic and prognostic biomarker. In the same study, plasma p-tau181 showed an early increase in participants who progressed to AD dementia, but not in those who progressed to non-AD dementia. In a separate cohort of 63 individuals with neuropathological data, antemortem plasma p-tau181 was associated with AD neuropathology in autopsy. These results suggest the potential use of p-tau181 as first line of testing to identify patients likely to be tau-positive when tested by PET or CSF biomarkers, either to distinguish AD from other non-AD neurodegenerative diseases in cases with mild to moderate dementia or predict future development of AD in cases with MCI (Janelidze et al., 2020).

In a different study, Barthélemy et al. (2020) suggested that p-tau217 in plasma could be more accurate than p-tau181 in detecting abnormal CNS tau metabolism. Estimated concentrations of t-tau (1–20 pg/ml) and p-tau-181 (sub pg/ml), as reported by immunoassays in plasma, are low and present a challenge for measuring plasma tau. Moreover, p-tau217 in the CSF is approximately five times less abundant than p-tau181. The sub pg/ml range estimated for p-tau-217 measurement is far below the concentration of currently monitored plasma biomarkers (Barthélemy et al., 2020). To address this problem, Barthélemy et al. (2020) designed an enrichment protocol to purify and concentrate plasma tau from 20 ml of plasma to 25 μl of final extract, leading to an enrichment factor of ∼800 times. Both plasma and CSF were analyzed for t-tau and p-tau peptides with a highly sensitive and resolute mass spectrometer equipped with a nano-flow capillary liquid chromatography device interfaced with nano-electrospray ionization (Barthélemy et al., 2020). Using this technique, no correlation was found between CSF and plasma t-tau levels as previously reported by immunoassays, suggesting that plasma t-tau level is not a biomarker for amyloid status and AD dementia (Barthélemy et al., 2020). In a cross-sectional study that included 1,402 participants, Palmqvist et al. (2020) found that plasma p-tau217 was able to differentiate AD from other neurodegenerative diseases with an accuracy of 0.89 and 0.96 in a neuropathological and clinically defined cohort, respectively. However, plasma p-tau217 performance was not significantly better than that of established CSF or PET biomarkers (Palmqvist et al., 2020).

### Other Potential Blood-Derived Markers

#### Enzyme β-Secretase 1

Enzyme β-secretase 1 (BACE1) is responsible for the first cleavage step in the production of Aβ peptides from APP. Based on ELISA methods, the activity of BACE1 has consistently been detected to be increased in plasma of patients with MCI and AD compared with healthy control groups. In a recent study, Shen et al. (2018) found a significant increase of plasma BACE1 activity in patients with MCI (by 53.2%) and probable AD (by 68.9%). In addition, individuals with MCI that progressed to AD over a 3-year follow-up period exhibited higher BACE1 activity than patients with MCI that remained cognitively stable after 3 years. BACE1 activity in plasma also showed sensitivity of 84% and specificity of 88% for the patients, which indicates the potential value of this marker in primary care and clinical trial settings as a prognosis biomarker of AD (Shen et al., 2018).

In contrast, a previous longitudinal study on a 2-year follow-up on platelets showed low BACE1 discriminator value for individuals who converted from MCI to AD, with an AUC of 0.64 (*p* = 0.04)(McGuinness et al., 2015). However, platelet BACE1, combined with A-disintegrin and metallopeptidase 10 (ADAM10) and presenilin 1 (PSEN1), showed high diagnostic value (AUC = 0.9) and high sensitivity (88.9%) for discriminating patients with AD from controls (Bram et al., 2019). These results suggest the potential use of BACE1 as a diagnostic biomarker in AD platelets; nevertheless, further validation studies on this subject are necessary.

#### Neurofilament Light

Neurodegeneration refers to any pathological condition that results in progressive loss of structure or function of neurons. Neurofilament light chain (NF-L) is a cytoplasmic neurofilament expressed in axons, where it plays an essential role in axonal homeostasis and synaptic transmission. In AD, abnormal aggregation and alterations of neurofilaments have been reported with increased levels of plasma and the CSF NF-L subunit proportional to axonal damage. NF-L can be measured with immunoassays in CSF and plasma and may serve as a promising diagnostic and prognostic neurodegeneration marker for AD (Mattsson et al., 2017; Lewczuk et al., 2018). High plasma NF-L levels appear to correlate with poor cognition and brain atrophy, and distinguish between AD, MCI, and healthy controls in sporadic AD, with higher values among subjects with MCI associated with more rapid brain atrophy. A recent study on an ADNI cohort found a correlation between plasma and CSF NF-L (Mattsson et al., 2017), suggesting that blood measurements reflect brain pathophysiology. Plasma NF-L was reported to increase in patients with MCI and AD compared with healthy controls and distinguish patients with AD from control with high accuracy (AUC = 0.87), a value comparable to plasma Aβ42/Aβ40 ratio results (Mattsson et al., 2017). Lewczuk et al. (2018) also found an increase in NF-L plasma levels and a significant association with age in patients with MCI and AD. High accuracy (AUC = 0.82), sensitivity (84%), and specificity (78%) were also reported in the study for AD diagnosis. Taken together, these findings suggest the potential usefulness of plasma NF-L as a non-invasive and cost-effective biomarker for early detection and prognosis of AD. However, plasma levels of NF-L are not specific for AD; increased plasma NF-L levels are found in neurodegenerative diseases such as frontotemporal dementia. Therefore, NF-L concentration measures in plasma might be of value as a first neurodegeneration screening tool in primary care settings of patients with cognitive disturbances.

### Plasma Protein Panels

Given the heterogeneous nature of AD, a combination of markers might perform better for the diagnosis, prognosis, and characterization of the disease than individual biomarkers. Several protein panels have been designed for case vs. control AD identification to estimate disease-related phenotypes and disease progression (e.g., Aβ deposition, cognitive decline, and brain atrophy), replace or aid validated diagnostic biomarkers (e.g., CSF Aβ42 status and Aβ PET), and improve the diagnostic accuracy for AD. Non-protein analytes (e.g., microRNAs, amino acids, and lipids) have also shown diagnostic or prognostic potential; however, independent and large-scale validation studies are required. In the following sections, we review recent developments in the field of protein blood-based biomarker panels for early diagnosis and prognosis of AD.

### Biomarker Panels With Prospect for Improving Current Diagnostic Assessment

Previous studies have shown a correlation among cortical Aβ accumulation with plasma Aβ42/Aβ40 rate, APOE phenotype (Tateno et al., 2017), and age (Tateno et al., 2017; Verberk et al., 2018; Chatterjee et al., 2019; Schindler et al., 2019; Doecke et al., 2020). Verberk et al. (2018) identified an association among plasma Aβ42/40, age, and APOEε4 status, with higher prediction accuracy after combining these markers (AUC = 0.83). More recently, the same authors reported another panel that better identified positive amyloid PET status [AUC = 0.88 (95% CI: 83–93%), Sn = 82%, Sp = 86%]. This panel included Aβ42/40 and glial fibrillary acidic protein (GFAP) alongside age and *APOE* (Verberk et al., 2020). Schindler et al. (2019) also found a correlation between plasma levels of Aβ42/Aβ40 and age, APOEε4 status, and gender. Their model predicted amyloid PET status with high accuracy (AUC = 0.94), proving its potential use as a screening tool for brain amyloidosis in cognitively normal individuals (Schindler et al., 2019). In a more recent study, Doecke et al. (2020) demonstrated a plasma assay’s reproducibility with the combination of Aβ42/40, age, gender, and APOEε4 to predict amyloid PET status with an AUC ranging from 0.88 to 0.91 over three time points. In addition, plasma P-tau181, when combined with Aβ42/Aβ40, was able to identify cerebral Aβ pathology with high accuracy (AUC = 0.84) (Janelidze et al., 2020). Similarly, the combination of plasma Aβ24, Aβ40 peptides, and *APOE*, measured using Elecsys immunoassays, predicted Aβ status in all stages of AD with AUC = 0.85–0.86, which could represent a reduction of 30–50% of PET costs in an AD trial screening scenario (Palmqvist et al., 2019).

Brain-derived exosomes have been proposed as potential biomarkers of Alzheimer’s disease. Jia et al. (2019) explored the diagnostic capacity of blood exosomal Aβ42, t-tau, and p-tau181 to predict AD in the asymptomatic stage. Significant differences in blood concentrations of exosomal Aβ42, t-tau, and p-tau181 in AD, amnestic mild cognitive impairment (aMCI), and control groups were found (*R*2 = 0.54–0.7). Differences were positively correlated with their CSF levels, suggesting these candidate’s potential clinical values for AD diagnosis. Additionally, they studied the diagnostic power of exosomal Aβ42, t-tau, and p-tau181 combined. The panel yielded higher diagnostic efficiency (AUC = 0.85–0.98) than individual blood and CSF markers (Jia et al., 2019). A similar study measuring AD-related proteins in extracellular vesicles (EVs) showed reduction of t-tau levels in subjects with MCI and patients with mild or moderate AD compared to controls. The levels of APP in EVs were also decreased in MCI and mild AD but increased in patients with severe AD compared to the controls. Concentrations of Aβ42 and p-tau181 in pEVs were higher in patients in the moderate AD stage than in the control group. ROC analysis revealed that APP concentration and the ratio of p-tau181/t-tau in EVs could distinguish controls from patients with MCI with significant accuracy [AUC = 0.923 ± 0.057 (*p* < 0.001) and 1 (*p* < 0.01), for APP and p-tau181/t-tau ratio, respectively]. Additionally, p-tau181/t-tau ratio seemed to be a good candidate marker to discriminate control from AD [AUC of 0.823 ± 0.091 (*p* < 0.05)] and MCI from AD [AUC of 0.87 ± 0.073 (*p* < 0.05)] (Perrotte et al., 2020). A more recent study on EV and plasma showed diagnostic capabilities of CLEC1B/CCL11 ratio in EVs (AUC = 0.95, 95% CI = 0.86–1, *p* = 0.001) and TGFα/CCL20 ratio in plasma (AUC = 0.96, 95% CI = 0.88–1, *p* = 0.001), measured using a proximity extension assay (Ellegaard Nielsen et al., 2020). However, it is important to mention the remarkably small number of subjects included in the aforementioned studies [*N* = 30: *n*(AD) = 10, *n*(MCI) = 1 0, *n*(HC) = 10; and *N* = 60: *n*(HC) = 12, *n*(MCI) = 12, *n*(mild AD) = 12, *n*(moderate AD) = 12, *n*(severe AD) = 12], respectively, suggesting that the reproducibility of these findings need to be tested on larger cohorts for further validation.

Westwood et al. (2020) identified putative plasma markers in relation to *in vivo* AD pathology and disease progression using a range of proteomic approaches, including mass spectrometry, SOMAscan, and immunocapture. Westwood et al. (2020) determined that the combination of seven plasma proteins (FCN2, B2M, APOE, A1AT, CC4, cathepsin D, and CFI), along with age, was the most replicable set of markers predicting *in vivo* brain amyloid pathology (AUC = 0.74) after testing 31 different candidate biomarkers on a large multi-center cohort of individuals with high and low amyloid burden. Using a machine learning approach, Eke et al. (2020) proposed a biomarker signature including eotaxin-3 (EOT3), apolipoprotein C1 (APOC1), glycoprotein hormone alpha chain (CgA), Aβ42, and APOE ε4 status, which strongly predicted CSF Aβ42 (AUC = 0.84; Sp = 0.62; Sn = 0.82). This combination yielded a positive predictive value (PPV) of 0.81 and a negative predictive value (NPV) of 0.64, and offered a less invasive and inexpensive solution to detect amyloidosis as the first step in a multistage diagnostic examination (Eke et al., 2020). These results are consistent with a previous random forest model developed by Goudey et al. (2017), which included same biomarkers with equal accuracy (AUC = 0.84) and similar sensitivity (0.78) and specificity (0.73), suggesting the potential of Aβ42, APOE, CgA, and EOT3 in combination with the APOE ε4 carrier for prediction of CSF Aβ42 levels.

### Biomarker Panels for Alzheimer’s Disease Characterization, Diagnosis, and Prognosis

Based on the complexity of AD’s pathogenesis, multivariate biomarker panels associated with various biological pathways may diagnose AD with greater accuracy than individual markers. Therefore, in recent years, several biomarker combinations have been proposed for characterization, discrimination among AD, MCI, and healthy controls, and prognosis of the disease.

Primary care is one of the most essential practices for early diagnosis of Alzheimer’s disease in the elderly, helping determine whether a patient should be referred to a memory clinic. Thus far, biomarkers studies have focused on diagnosing the disease, with less attention to screening tools needed for broad-based implementation in primary care settings. Jammeh et al. (2016) identified a panel of six blood biomarkers that detected AD with a sensitivity of 85.4% and a specificity value of 78.6%. The panel included A1M, A2M, alpha-1 Antitrypsin (AAT), APOE, complement C3, and the serine/threonine phosphatase PPP, in combination with age as a covariate; which may be used as a cost-effective point-of-care AD diagnostic tool (Jammeh et al., 2016). Using a machine learning approach, Eke et al. (2018) also proposed using A1M, A2M, and complement C3 in combination with immunoglobulin M (IgM), Tenascin C (TNC), and APOE genotype as covariate. The panel showed high accuracy (85%) and AUC (0.89) for the diagnosis of AD in an ADNI cohort (Eke et al., 2018). A third study identified a panel including serum Aβ clearance protein complement C3, transthyretin (TTR), and APOA, which achieved an AUC of 0.89% and high sensitivity (83%) and specificity (90%) for detecting late-MCI (Liu et al., 2019).

Brain-derived neurotrophic factor (BDNF) is a member of the neurotrophin family of growth factors with an essential role in brain development, neuroplasticity, and neuronal survival. Plasma levels of BDNF have been previously identified as reduced in AD, which might contribute to cognitive dysfunction. Janel et al. (2017) reported decreased levels of BDNF in the plasma of patients with AD, and diagnostic accuracy of 0.754. In the study, BDNF showed a positive correlation with DYRK1A (*P* < 0.05), which individually had a better performance for AD diagnosis (AUC = 0.847), and a negative correlation with homocysteine (Hcy) in plasma. Taken together, these markers in a panel showed high accuracy (93.3%), sensibility (0.952), and specificity (0.889) for diagnosing AD (Janel et al., 2017). In a study on a Han Chinese population, Cheng et al. (2018) found an eight-protein panel composed of BDNF, angiotensinogen (AGT), insulin-like growth factor binding protein 2 (IGFBP-2), osteopontin (OPN), cathepsin D, serum amyloid P component (SAP), complement C4, and transthyretin (TTR) to have a high diagnostic score for AD vs. healthy controls (AUC = 0.958; Sn = 0.867; Sp = 0.881) (Cheng et al., 2018). Schipke et al. (2019) also identified a panel that included BDNF in combination with IGF-1, VEGF, TGF-β1, MCP-1, and IL-18 to be robust (AUC = 0.94; Sn = 76%; Sp = 95%; accuracy = 85%) and reproducible in serum samples using ELISA kits. These results support the role immunoregulatory proteins might play in AD and interestingly showed high accuracy of various panels including BDNF. Further validation of these protein’s specific role and studies on longitudinal and larger cohorts would be useful.

Immune response biomarkers have reported associations with cognitive performance, cognitive decline, and clinical progression of AD. In a recent study, Yu et al. (2017) identified 17 potential serum biomarkers, such as cytokines, chemokines, and growth and metabolic factors. Based on a random forest mathematical algorithm, Yu et al. (2017) also proposed an eight-protein panel for AD screening with a diagnostic accuracy or AUC of 0.99 (Sp = 88.6%; Sn = 97.7%; *p* < 0.05). The panel included cytokines (IL-13, IL-1α, CXCL10, IL-3, and TNF-α) and growth factors (leptin, resistin, and PAI-1), which support the occurrence of immune and neuroinflammatory processes in the development and progression of AD (Yu et al., 2017). A previous study on pro-inflammatory cytokines showed CXCL10 and IL-13 to have high diagnostic accuracy levels with 100% sensitivity and specificity for diagnosing AD (Mohd Hasni et al., 2017). In a previous study, Burnham et al. (2014) have proposed a six-plasma protein panel to estimate neocortical Aβ deposition in the Australian Imaging, Biomarker, and Lifestyle (AIBL) cohort. The signature included Aβ42, CXCL13, IgM-1, IL-17, PPY, and VCAM-1, combined with age, and it showed sensitivity and specificity of 80 and 82%, respectively. Validation of the ADNI cohort resulted in 79 and 76% of sensitivity and specificity, respectively (Burnham et al., 2014). The same group published a 54-month follow-up study after identifying disease progression; cognitively normal individuals considered at risk by the panel progressed to having MCI or AD (odds ratio = 2.4) in comparison to those considered not at risk. Participants with MCI considered at risk by the signature progressed to having AD (odds ratio = 12.3) in comparison to those considered not at risk (Burnham et al., 2016).

IL-10 and IL-12/23p40 have been proposed to predict Aβ deposition in healthy controls from the AIBL cohort (Pedrini et al., 2017). IL-10 and IL-12/23p40, when combined with age, sex, and APOE ε4 status, reported an AUC of 0.805 when evaluating healthy controls at 54 months and an AUC of 0.802 at 18 months. Pedrini et al. (2017) found proteins eotaxin-3, leptin, and PYY to be altered in individuals with AD in correspondence with their APOE ε4 status. More recently, Boccardi et al. (2019) observed that a joint expression of three proteins (IFN-α2, IL-1α, and TNFα) could discriminate HC from AD with an accuracy of 65.24%. These results highlight potential biomarkers for early identification of at-risk individuals developing AD and early diagnosis of the disease.

### Other Blood-Based Biomarker Panels

It has been shown that synaptic damages occur in the asymptomatic stage of Alzheimer’s disease. Brain tissues of patients with AD exhibit decreased synaptic proteins such as growth-associated protein 43 (GAP43), neurogranin, synaptotagmins, Rab3A, and synaptosome-associated protein 25 (SNAP25). Moreover, increased expressions of GAP43, neurogranin, SNAP25, and synaptotagmin 1 have also been observed in the CSF of patients with AD, indicating their potential as markers of the disease. SNAP25 has also shown high diagnostic accuracy (AUC = 0.826) and sensitivity (87.5%) in serum exosomes (Agliardi et al., 2019). A recent Chinese study revealed that exosomal SNAP25, GAP43, neurogranin, and synaptotagmin-1, combined with APOE ε4 status, improved diagnostic accuracy (AUC = 0.88), acting as a useful biomarkers panel for the prediction of AD five to seven years before cognitive impairment. Concentrations of these exosomal markers were highly correlated with those in the CSF; however, the study showed poor significant accuracy in detecting preclinical AD of the proteins individually, which might be due to minimal alterations in exosomal synaptic proteins in this stage of the disease (Jia et al., 2021).

## Future Directions

Understanding AD’s clinical and pathophysiological heterogeneity, and constructing a comprehensive model for disease progression have become a major challenge in clinical research. Current established biomarkers capture three main pathophysiological events (amyloidosis, tauopathy, and neurodegeneration); however, they fail to explain the heterogeneity of individual clinical trajectories and disease biomarker variance. Studies have shown that neurodegeneration can be expressed differently across AD histopathological subtypes, and that diagnosed patients with probable AD present differences in impaired cognitive domains (Scheltens et al., 2016; Ferreira L.S.S. et al., 2018; Jia et al., 2021). Furthermore, several studies have shown AD-related neuropathologies, such as neurofibrillary tangles and tau pathology, upon autopsy on cognitively normal individuals. Whether these pathologies reflect processes related to normal aging or preclinical AD is still in debate. However, using the ATN research framework described by the NIA-AA, individuals without cognitive impairment but with elevated Aβ markers are on the AD *continuum* and potential biomarker disclosure should be shared with those individuals. Newly proposed biomarkers and biomarker panels should be more extensively studied in this context to characterize the clinicopathological heterogeneity of AD and disambiguate it from normal aging.

The search for accurate blood biomarkers for AD diagnosis and prognosis is a broad topic in continuous development. During this review’s editorial process, we had taken notes of recent critical advances in blood protein biomarker panels. For example, the combination of plasma measurements Aβ42/40, p-tau217, and NF-L predicted change in cognition and subsequent AD dementia (AUC = 0.82, 95% CI [0.77–0.91], *P* < 0.0001), showing that plasma biomarkers are linked to AD-related changes in the cognitively unimpaired elderly and can significantly reduce sample sizes needed to run clinical trials (Cullen et al., 2021). Plasma p-tau, in combination with cognitive tests and APOE genotyping, seems to greatly improve the diagnostic prediction of AD and predict future metabolic dysfunction (Lussier et al., 2021; Palmqvist et al., 2021). Other recent studies confirmed that plasma GFAP levels are elevated in cognitively normal older adults at risk of AD (Chatterjee et al., 2021) and in Aβ + CU individuals. When this glial protein was taken together in a biomarker panel with Aβ42/Aβ40 ratio, it distinguished Aβ+ accurately from Aβ− (AUC = 0.92), highlighting the potential use of this plasma biomarker panel to contribute to AD diagnosis (Chatterjee et al., 2021). Nevertheless, we believe that the remaining challenges to make testing for AD pathology more widely accessible are needs for: (i) more extensive validation of current advances in blood biomarkers, and (ii) development of new methodologies, since the existing techniques are still complex and expensive for large-scale implementations and screening purposes in low- to middle-income countries.

Use of several tau isoforms may provide additional information on AD stages leading to highly accurate diagnostic blood tests. Plasma p-tau231 has been recently proposed as a potential blood-based biomarker of emerging AD pathology with clinical utility as a rapid screening test, and in clinical trials to targeting vulnerable population under the threshold of Aβ positivity and early tau deposition. Ashton et al. (2021) developed a highly sensitive assay for quantification of p-tau231, which exhibited high performance for differential diagnosis of AD. P-tau231 was capable of differentiating AD from Aβ–cognitively unimpaired individuals (AUC = 0.92–0.94), AD from patients with other neurodegenerative disorders (AUC = 0.93), as well as AD from Aβ-MCI individuals (AUC = 0.89) with high accuracy (Ashton et al., 2021). Plasma p-tau231 was able to identify the clinical and neuropathological stages of AD progression and detected very early stages better than p-tau181 and Aβ PET. So far, it is the only blood-based biomarker capable of differentiating individuals across the entire Braak stage spectrum, and it seems to be a good indicator of early AD tau pathology, proving its potential value for early treatment and accurate prognosis.

Furthermore, subtle differences in performance across plasma phosphorylated tau species and platforms for predicting amyloid and tau PET and MRI measures have also been recently reported (Bayoumy et al., 2021; Mielke et al., 2021). Mielke et al. (2021), in a head-to-head comparison of plasma p-tau species measured by Simoa and MSD platforms, reported a stronger association of MSD p-tau181 and p-tau217 with MRI AD measures than with Simoa p-tau181 and p-tau231. MSD p-tau181 (AUC = 0.8) and p-tau217 (AUC = 0.81) also reported better results than Simoa p-tau181 (AUC = 0.7) for predicting entorhinal cortex tau PET among CU participants (Mielke et al., 2021). In another study, using electrochemiluminescence-based assays, plasma p-tau217 (AUC = 0.93 [0.91–0.96]) better differentiated patients with AD from other neurodegenerative diseases compared to p-tau181 (AUC = 0.91 [0.88–0.94]). P-tau217 also seemed to be a better predictor of amyloid PET (AUC = 0.91 [0.88–0.94]) than p-tau181 (AUC = 0.89 [0.86–0.93]) and tau PET binding in the temporal cortex. Nevertheless, both p-tau217 and p-tau181 showed their usefulness as screening tools with excellent diagnostic performance in identifying individuals with AD amyloid and tau pathologies (Thijssen et al., 2021). Moreover, a head-to-head comparison of plasma amyloid assays for detecting Aβ brain pathology has also been recently published (Janelidze et al., 2021).

In addition, a high percentage of individuals diagnosed with probable AD dementia show co-occurrence of multiple brain pathologies that overlap with other diseases (such as diabetes mellitus, cardiovascular, and cerebrovascular diseases) (Ramirez et al., 2017; Boyle et al., 2018; Mnn et al., 2018). Several studies have revealed shared common pathogenic mechanisms between AD and type 2 diabetes (such as insulin signaling and glucose metabolism impairment, inflammatory pathways, and neuronal stress signaling), which lead to neurodegeneration, memory deficits, and cognitive decline (Gm and Sa, 2007; de la Monte, 2017; Ferreira D. et al., 2018; Mnn et al., 2018; Rad et al., 2018; Wijesekara et al., 2018; Hölscher, 2019). The discovery of brain-specific insulin signaling deficit and insulin resistance in AD pathogenesis has led to the recent denomination of AD as “Type-3-Diabetes” (Kandimalla et al., 2017; Leszek et al., 2017; Tt et al., 2020). Several recent studies have investigated and reviewed peripheral biomarkers common to both diseases with promising results (Movassat et al., 2019; Kubis-Kubiak et al., 2020; Pekkala et al., 2020; Pereira et al., 2021). However, although extensive research has been conducted to understand underlying biological mechanisms that link AD and diabetes, the vast majority of evidence is based on animal models and observational clinical studies. Further studies on large cohorts of individuals at risk for dementia, and with longitudinal data are needed to further investigate the associations between AD and diabetes mellitus. In addition, questions such as how other brain diseases might influence AD progression and derived therapeutic strategies should be explored more extensively.

## Conclusion

Alzheimer’s disease, as the primary cause of dementia in the elderly and affecting millions of people worldwide, is of unquestionable concern. Recent advances in neuroimaging and CSF-based technologies have a significant impact on the detection and characterization of this disease. However, non-invasive and inexpensive assessments for AD diagnosis, prognosis, or primary care prescreening tools are urgently needed. Blood-based biomarker tools have been suggested as less invasive and cost-effective alternatives that meet scalability requirements for broad population-based screenings.

As shown in our review, a wide variety of blood-based biomarker panels have been recently examined for early AD diagnosis and prediction of MCI conversion to AD. Protein biomarker panels outperformed single candidate markers in detection of the disease. Aβ42/Aβ40 ratio in plasma in combination with age, APOEε4 status, and gender, seems to be a promising panel for the prediction of amyloidosis due to AD; thus, it may be of use as a less invasive and cost-effective screening tool. The combination of plasma Aβ42/Aβ40, p-tau217, and p-tau181 seems to be a potential non-invasive and cost-effective biomarker for diagnosing AD, while other individual markers like plasma p-tau181, NF-L, and BACE1 may be used as markers of disease progression and neurodegeneration. Further validation studies on the proposed biomarkers in larger cohorts from various populations and longitudinal studies are needed.

## Data Availability Statement

The original contributions presented in the study are included in the article/Supplementary Material, further inquiries can be directed to the corresponding author.

## Author Contributions

AH-S, KL-A, GG-N, JL-G, and PV-S: study concept and design. AH-S, SB, KL-A, and JL-G: acquisition, analysis, or interpretation of the data. AH-S, JL-G, and PV-S: drafting of the manuscript. PV-S and GG-N: project administration. PV-S, JL-G, JB-A, and GG-N supervised the study. All authors had full access to all the data of the study and they took responsibility for the integrity and accuracy of the analysis and results, and critically revised the manuscript.

## Conflict of Interest

The authors declare that the research was conducted in the absence of any commercial or financial relationships that could be construed as a potential conflict of interest.

## Publisher’s Note

All claims expressed in this article are solely those of the authors and do not necessarily represent those of their affiliated organizations, or those of the publisher, the editors and the reviewers. Any product that may be evaluated in this article, or claim that may be made by its manufacturer, is not guaranteed or endorsed by the publisher.
