# Supplementary material for: Diagnostic Accuracy of Blood-Based Biomarker Panels: A Systematic Review
Source: Front Aging Neurosci. 2022 Mar 11;14:683689. doi: 10.3389/fnagi.2022.683689 (PMC8963375; doi:10.3389/fnagi.2022.683689)
Supplement: Supplementary file 1 [file Table_1.pdf]

Supplementary Material

Supplementary Table 1. A detailed summary of study characteristics included in the systematic review

Study	*Study Design*	*Cohort(s)*	*Sample size*	*Tissue*	*Proposed blood biomarker(s)*	*Analytical technique*	*Statistics*	*Type of biomarker(s)*
McGuinness et al., 2015 (1)	Longitudinal study (2-year follow-up)		N = 182<br>MCI (97), CN (85)	Platelet	BACE1	BMG Labtech FLUOstar OPTIMA	AUC=0.64 (p=0.04)	Prognostic
Burnham et al., 2016 (2)	Longitudinal study (54-month follow-up)	AIBL	N = 817<br>CN (585), MCI (74), AD (158)<br>Validation group: CN (227), MCI (11), AD (5)	Plasma	Aβ42 + CXCL13 + IgM-1 + IL-17 + PPY + VCAM-1	Luminex human discovery 151MAP pane	Sn=80%; Sp=82%<br>Validation cohort: Sn=79%; Sp=76%	Diagnostic
Janelidze et al., 2016 (3)	CC	BioFINDER	N = 719<br>CN (274), SCD (174), MCI (214), AD (57)	Plasma	Aβ40, Aβ42	Simoa platform	Aβ42: AUC (CSF) = 0.655, AUC (PET) = 0.604<br>Aβ42/Aβ40 ratio: AUC = 0.683, AUC (PET) = 0.621	Diagnostic
Jammeh et al., 2016 (4)	CC	ADNI	N = 157<br>AD (106), CN (51)	Plasma	A1M + A2M + C3 + AAT + APOE + PPP	-	AUC=0.85, Sn=85.4%, Sp=78.6%, accuracy = 83.6%	Diagnostic
Yu et al., 2016 (5)	CC		N = 90<br>AD (50), CN (40)	Serum	VEGF + sCD40L	Immunomagnetic beads assay	AUC=0.58 (95% CI: 0.775-0.941)	Diagnostic
Zheng et al., 2016 (6)	Longitudinal study (2-years follow-up)		N = 254<br>CN (90), MCI-AD (76), MCI-MCI (88)	Serum	HCY + BDNF + APOEε4	ELISA and microparticle-enzyme-immunoassay	Sn=85.0%, Sp=86.0%, PPV=0.93, NPV=0.73%	Predictive for early diagnosis
An et al., 2017 (7)	CC		N = 171<br>AD (24), CN (29)	Plasma	Aβ oligomers	Multimer detection system	Sn=83.3%, Sp=90.0%	Diagnostic

Goudey et al., 2017 (8)		ADNI	N = 566<br>Training: MCI (198), AD (102), CN (58)<br>Validation: MCI (198), AD (10)	Plasma	Aβ42 + APOE + CgA + EOT3 + APOEε4 status	-	AUC=0.84, Sn=78%, Sp=0.73%	Predictive of CSF Aβ42 status
Janel et al., 2017 (9)	CC		N = 140<br>Cohort (M): CN (20), AD (69)<br>Cohort (P): CN (25), AD (26)	Plasma	DYRK1A + BDNF + HCY	Solid-phase immobilized epitope-immunoassay, ELISA, and fluorimetric high-performance liquid chromatography	AUC=0.933; Sn=95.2%; Sp=88.9%	Diagnostic
Mattsson et al., 2017 (10)	Longitudinal study (6.5-years follow up)	ADNI	N = 570<br>CN (193), MCI (197), AD (180)	Plasma	NFL	Ultrasensitive single-molecule array (Simoa) platform	AUC=0.87	Diagnostic and prognostic
Mohd Hasni et al., 2017 (11)	CC		N = 78<br>AD (39), CN (39)	Serum	IL-13 + CXCL10	Procarta Multiplex Cytokine and ELISA assay	AUC=1 (95% CI); Sp=100%; Sn=100%	Diagnostic
Pedrini et al., 2017 (12)	Longitudinal study (18- and 54-months follow-up)	AIBL	N = 665<br>18 months: CN (559), MCI (39), AD (67)<br>54 months: CN (528), MCI (51), AD (86)	Plasma	IL-10 + IL-12/23p40	Multiplex assay panel	18 months: AUC=0.802<br>54 months: AUC=0.805	Early diagnosis
Popp et al., 2017 (13)	CC		N = 120<br>CN (48), MCI (72)	Serum	bFGF + CRP + IL-16 + sFLT-1 + sICAM-1 + Tie-2 + VEGF-C + VEGF-D	V-Plex Neuroinflammation Panel 1	AUC=0.89 (0.81 - 0.95)	Classification for AD pathology
Tateno et al., 2017 (14)	CC		N = 117<br>ApoE4 (28), non-ApoE4 (89)	Plasma	Aβ42/Aβ40 + APOEε4	Sandwich ELISA assay	APOEε4 (+): AUC=0.519, Sn=63.6%, Sp=52.9%<br>APOEε4 (-): AUC=0.648,	Diagnostic

							Sn=92.9%, Sp=45.9%, PPV=44.1%, NPV=93.3%	
Wang et al., 2017 (15)	CC		N = 61<br>AD (24), CN (37)	Plasma	Aβ oligomers	Multimer detection system	AUC=0.844, Sn=78.3%, Sp=86.5%	Diagnostic
Yu et al., 2017 (16)	CC		N = 312<br>AD (156), CN (156)	Serum	IL-13 + IL-1α + CXCL10 + IL-3 + TNFα	Immunomagnetic beads assay	AUC=0.58 (95% CI: 0.775-0.941)	Diagnostic
Chen et al., 2018 (17)	CC		N = 126<br>AD (96), CN (30)	Serum	APP + NCAM + Aβ40 + Aβ42	ELISA assay	AUC=0.997, Sn=98.5	Diagnostic and prognostic
Cheng et al., 2018 (18)	CC		N = 199<br>AD (98), CN (101)	Plasma	BDNF + AGT + IGFBP-2 + OPN + cathepsin D + SAP + C4 + TTR	Luminex xMAP	AUC= 0.958, Sn=86.7%, Sp=88.1%, Accuracy=87.4%	Diagnostic
de Rojas et al., 2018 (19)	CC	FACEHBI	N = 200	Plasma	Aβ42/Aβ40	Sandwich ELISA assay	AUC=0.681, Sn=83%, Sp=59.1%	Predictive of amyloid PET
Eke et al., 2018 (20)	CC	ADNI	N = 166<br>AD (108), CN (58)	Plasma	A1M + A2M + C3 + IgM + TNC	-	AUC=0.89, Sn=86.5%, Sp=82.1%, Accuracy=85%	Diagnostic
Lewczuk et al., 2018 (21)	CC		N = 140<br>MCI (25), AD (33), CN (41)	Plasma	NFL	Simoa assay	Age (-): AUC=0.853, Sn=84%, Sp=0.78%, accuracy=82%<br>Age (50-80): AUC=0.920, Sn=61%-91%, Sp=89%- 20%, accuracy=84%-80%	Diagnostic (nonspecific)
Mielke et al., 2018 (22)	CC	MCSA, ADRC	N = 269<br>CN (172), MCI (57), AD (40)	Plasma	p-tau181, p-tau181 + APOEε4+	MSD	AUC (p-tau181) = 0.803<br>AUC (p-tau181 + APOEε4+) = 0.860	Predictor of elevated brain Aβ
Nabers et al., 2018 (23)	Longitudinal study	BioFINDER, ESTHER	N = 385<br>BioFINDER:<br>PET+ (36), PET- (37)	Plasma	Aβ secondary structure	Vertex 70V FTIR-spectrometer	AUC (BioFINDER) = 0.78, Sn = 69%, Sp = 86%	Diagnostic

			ESTHER:<br>AD (65), CN (247)				AUC (ESTHER) = 0.80, Sn = 71%, Sp = 91%	
Nakamura et al., 2018 (24)	CC	NCGG, AIBL	NCGG = 121, AIBL = 252	Plasma	Aβ42 + APP/Aβ42 + Aβ42/Aβ40	Immunoprecipitation coupled with mass spectrometry	Discovery: AUC=0.967<br>Validation: AUC=0.941<br>Accuracy=90%	Predictive of amyloid PET
Shen et al., 2018 (25)	Longitudinal study (3-years follow-up)		N = 224<br>Probable AD (75), MCI (96), ND (53)	Plasma	BACE1	Synthetic fluorescence substrate ELISA and Western blot	Sn = 84%, Sp = 88%	Prognostic
Verberk et al., 2018 (26)	Longitudinal study (3 ± 2 years follow-up)	SCIENCe project and Amsterdam Dementia Cohort	N = 248	Plasma	Aβ42/Aβ40 + APOEε4 status	Simoa Human Neurology 3-Plex A assay kit	AUC=0.83 (95% CI 0.77-0.89), Sn=76%, Sp=75%	Prescreener, diagnostic
Winston et al., 2018 (27)	CC		N = 137<br>CN (76), MCI (61)	Exosomes	Aβ42, NRGN, SYP, SYT, and SYNPO	ELISA assay	Aβ42: Sn=80.6%<br>NRGN, SYP, SYT, and SYNPO: Sn=100%	Diagnostic
Agliardi et al., 2019 (28)	CC		N = 42<br>AD (24), CN (17)	Exosomes	SNAP-25	Western Blot and ELISA	AUC=0.82, Sn=87.5%, Sp=70.6%	Diagnostic
Baldacci et al., 2019 (29)	CC		N = 78<br>Early AD (39), CN (39)	RBC	α-syn/Aβ, α-syn/tau	Immunoassays	AUC (α-syn/Aβ) = 0.76<br>AUC (α-syn/tau) = 0.72	Diagnostic
Boccardi et al., 2019 (30)	CC		N = 289<br>HC (87), MCI (73), AD (129)	Plasma	IFNa-2, IL-1, TNFa		AUC = 0.6524	Prognosis
Bram et al., 2019 (31)	CC		N = 40<br>AD (20), CN (20)	Platelet	ADAM10 + BACE1 + PSEN1	Western blot	AUC (ADAM10) = 0.9, Sn = 88.90%, Sp = 78.90%<br>AUC (PSEN1) = 0.80, Sn = 77.80%, Sp = 83.30%	Diagnostic

							AUC (ADAM10 + BACE1 + PSEN1) = 0.9	
Chatterjee et al., 2019 (32)	CC	KARVIAH	N = 95<br>SMC (72), CN (23)	Plasma	Aβ42/Aβ40 + APOEε4 status	Ultrasensitive Simoa assay	AUC=0.78	Predictive of amyloid PET
Chen et al., 2019 (33)	CC	HABS, UCL, UCSD	N = 151<br>Discovery cohort:<br>CN (19), MCI (21), AD (25)<br>Validation cohort:<br>CN (41), MCI (22), AD (23)	Plasma	Tau	ELISA and Simoa assay	Discovery cohort:<br>MCI vs. Con: AUC=0.79<br>AD vs. Con: AUC=0.75<br>Validation cohort:<br>MCI vs. Con: AUC=0.88<br>AD vs. Con: AUC=0.96	Diagnostic
Guzmán-Martínez et al., 2019 (34)	CC	FACEHBI	N = 51<br>AD (36), CN (15)	Platelet	Alz-tau® (HMW/LMW tau ratio)	SDS-PAGE and Western Blot	AUC=0.756, Sn=71.43%, Sp=69.23%	Diagnostic
Han et al., 2019 (35)	CC	KBASE	N = 407<br>CN (241), MCI (103), AD (63)	Plasma	AChE	ELISA and colorimetric assay	AChE + ApoE ε4: AUC=0.728-0.798<br>AChE activity + ApoE ε4: AUC=0.727-0.768	Predictor of cerebral Aβ deposition in cognitively normal individuals
Iulita et al., 2019 (36)	Longitudinal study (3 years follow-up)	LEAD	N = 107<br>pAD (28), MCI (30), SMI (30), CN (19)	Plasma	Aβ40 + Aβ42 + MMP-1 + MMP-3 + IL-8 + IL-10 + TNF- α	Multi-Spot V-PLEX	pAD: AUC=0.732<br>MoCA decline: AUC= 0.751<br>CAMCOG decline: AUC= 0.844	Diagnosis, prognosis
Jia et al., 2019 (37)	CC		N = 298<br>Discovery stage: AD (28), aMCI (25), CN (29)	Exosomes	Aβ42 + T-tau + P-T181-tau	Neuronal-derived exosomes immunoprecipitation and ELISA	Discovery cohort: AUC=0.86-0.97<br>Validation cohort: AUC=0.85-0.98	Predictive of CFS Ab42, T-tau, and P-T181-tau status

			Validation stage: AD (73), aMCI (71), CN (72)					
Kapogiannis et al., 2019 (38)	Longitudinal study (2 years follow-up)	BLSA, JHADRC	N = 414<br>BLSA cohort: MCI to AD (128), CN (222)<br>JHADRC cohort: AD (35), CN (29)	nEV	nEV concentration + nEV mean diameter + nEV proteins (Ttau, p-tau181, p-tau231, pY-IRS-1, and pSer312-IRS-1)	Neuronal-enriched extracellular vesicles immunoprecipitacion and ELISA	BLSA cohort:<br>Training set (n=161): AUC=0.896, Sn=81.8%, Sp=85.8%<br>Validation set (n=80): AUC=0.80, Sn=55.6%, Sp=88.7%<br>JHADRC cohort:<br>Training set: AUC=0.989, Sn=100%, Sp=94.7%<br>Validation set: AUC=0.767, Sn=91.7%, Sp=60%	Diagnostic
Li et al., 2019 (39)	CC		N = 84<br>AD (53), MCI (22), CN (9)	Plasma	Aβ42, Aβ40, T-tau	Simoa assay	Aβ42/Aβ40: AUC=0.77, Sn=82%, Sp=64%	Diagnostic
Liu et al., 2019 (40)	CC	Liu et al.	N = 63<br>CN (10), EMCI (26), LMCI (23), AD (4)	Serum	apoA-I + aC3/nC3 + TTR	Turbidimetric immunoassay and ELISA assay	AUC (apoA-I) = 0.77<br>AUC (aC3/nC3) = 0.79<br>AUC = 0.89, Sn=83%, Sp= 90%	MCI prognosis
Liu et al., 2019 (41)	Longitudinal study (2-year follow-up)		N = 92	Serum	ACT	ELISA and Western blot	AUC (6 months) = 0.688<br>AUC (12 months) = 0.839<br>AUC (24 months) = 0.887	aMCI diagnosis
Meng et al., 2019 (42)	CC		N = 58<br>AD (30), CN (28)	Plasma	Aβ oligomers	Multimer detection system	AUC=0.89, Sn=82.1%, Sp=90.0%	Diagnostic

Morgan et al., 2019 (43)	CC	AddNeuroMed, EMIF-AD MBD, DCR	N = 1580<br>Discovery cohort:<br>CN (259), MCI (199), AD (262)<br>Replication cohorts:<br>AD (193), MCI (494), CN (600)	Plasma	FB + FH + sCR1, MCP1 + EOT1<br>sCR1 + MCP1 + EOT1<br>FB + FH + age	Singleplex and multiplex assay, ELISA and V-plex electrochemiluminescence immunoassay	AD vs. Con: AUC (discovery/replication cohort) =0.74/0.81<br>AD vs. MCI: AUC (discovery/replication cohort) =0.74/0.67<br>MCI to AD: AUC=0.71	Diagnostic and prognostic
Nabers et al., 2019 (44)	CC	BioFINDER, ESTHER	N = 100<br>CN (61), AD (39)	Plasma	Aβ secondary structure	Spectrometry	BioFINDER: AUC=0.78, Sn=69%, Sp=86%<br>ESTHER: AUC=0.80, Sn=71%, Sp=91%	Diagnostic
Palmqvist et al., 2019 (45)	CC	BioFINDER and a German biomarker study for validation	N = 1079<br>CU, MCI, AD	Plasma	Ab42/Ab40, APOE+4<br>status, tau, NF-L	Elecsys immunoassays (Roche Diagnostics)	Cohort 1: 0.80–0.87;<br>Cohort 2: 0.86	Predictive of CSF Ab status
Park et al., 2019 (46)	CC		N = 76<br>CN (52), MCI (9), AD (15)	Plasma	T-tau/Aβ42	Simoa assay and xMAP technology	AUC=0.89, Sn=80%, Sp=91.43%	Predictive of tau pathology
Park et al., 2019 (47)	CC	KBASE	N = 254<br>CN (107), MCI (107), AD (40)	Plasma	Set I: LGALS3BP + ACE + gal-3<br>Set II: POSTN + ACE + VE-cadherin<br>Proposed biomarkers: LGALS3BP, CDH5, ACE, POSTN	Mass spectrometry and ELISA	Set I: AUC (PiB- vs. PiB+) =0.871, Sn=79%, Sp=84%<br>Set II: AUC (for MCI) = 0.836, Sn=68%, Sp=90%	Predictive of Ab deposition
Pérez-Grijalba et al., 2019 (48)	CC	AB255 study	N = 59<br>CN (39), MCI (20)	Plasma	Aβ42/Aβ40	ABtest kit	AUC=0.881, Sn=77.8%, Sp=87.5%, PPV=0.732, NPV=0.900	Predictive of Ab PET, diagnostic
Perrotte et al., 2019 (49)	CC		N = 95	Plasma	ApoJ, Klotho, protein carbonyls, circulating-proteosome	Immunoblotting, ELISA	*MCI vs. Con:*<br>AUC (ApoJ) = 0.8804	Diagnostic and prognostic

			CN (24), MCI (24), AD (47)				AUC (Klotho) = 0.8036<br>*AD vs. Con:*<br>AUC (ApoJ) = 0.8168<br>AUC (Klotho) = 0.7976<br>AUC (protein carbonyls) = 0.7528<br>AUC (circulating-proteosome activity) = 0.8440<br>*MCI vs. AD*:<br>AUC (protein carbonyls) = 0.7522<br>AUC (circulating-proteosome activity) = 0.8379	
Schindler et al., 2019 (50)	CC		N = 158	Plasma	Aβ42/Aβ40 + APOEε4 status	Immunoprecipitation, Mass spectrometry	AUC=0.94 (95% CI 0.90–0.97)	Diagnostic
Schipke et al., 2019 (51)	CC		N = 160<br>CN (79), AD (81)	Serum	BDNF + IGF-1 + VEGF + TGF-β 1 + MCP-1 + IL-18	ELISA assays	AUC=0.94, Sn=76%, Sp=95%, Accuracy=85%	Diagnostic
Whelan et al., 2019 (52)	CC	BioFINDER	N = 843<br>Control Aβ- (415), Control Aβ+ (142), MCI- Aβ- (50), MCI-Aβ+ (75), AD (161)	Plasma	OSM, MMP9, HAGH, CD200, AXIN1, uPA	Olink^{TM} ProSeek immunoassay	*AD vs. Con:*<br>AUC=0.94<br>*Prodromal AD vs. Con:*<br>AUC=0.78	Diagnostic
Yao et al., 2019 (53)	CC		N = 123<br>AD (54), CN (69)	Serum	GSN, BDNF, TIMP1, VLDLR, APLP2	ELISA and Western blot for validation	AUC (VLDLR) = 0.932<br>AUC (TIMP1) = 0.903	Diagnostic
Abate et al., 2020 (54)	CC	InveCe.Ab, PharmaCog/E-ADNI	InveCe.Ab: N = 264<br>PharmaCog/E-ADNI: N = 111	Plasma	Unfolded p53	-	AUC = 0.92	Predictive

Abe et al., 2020 (55)	Multicenter, CC	Abe et al.	N = 259<br>CN (100), MCI (60), and AD (99)	Serum	FBC + AHSG + FAC + PPC11	BLOTCHIP® - MS analysis	*MCI vs. Con:* AUC=0.662, Sn=72%, Sp=59%<br>*MCI vs. AD:* AUC=0.672, Sn=77%, Sp=62%<br>*AD vs. Con:* AUC=0.804, Sn=87%, Sp=65%	Diagnostic
Barthélemy et al., 2020 (56)	CC	SILK	Discovery cohort = 58<br>Validation cohort = 92	Plasma	p-tau-217, p-tau-181	Immunopurification (IP)-LC-MS	p-tau-217:<br>AUC (discovery) = 0.99<br>AUC (validation) = 0.92<br>p-tau-181:<br>AUC (discovery) = 0.98<br>AUC (validation) = 0.75	
Doecke et al., 2020 (57)	Longitudinal study (54 months follow-up)	AIBL	Month 18: n = 176<br>Month 36: n = 169<br>Month 54: n = 135	Plasma	Aβ42/Aβ40 + APOEε4	ELISA assays	AUC=0.88-0.913<br>AUC(HC)=0.808-0.898	Diagnostic
Eke et al., 2020 (58)	CC	ADNI	N = 358<br>CN (58), MCI (198), AD (102)	Plasma	Aβ42 + CgA + EOT3 + APOEε4	-	AUC=0.84, Sn=0.82, Sp=0.62, PPV=0.81, NPV=0.64	Predictive of CSF Aβ42
Ellegaard et al., 2020 (59)	CC		N = 30<br>AD (10), MCI (10), CN (10)	Plasma	CLEC1B/CCL11 ratio in EVs<br>TGFa/CCL20 ratio in plasma	Proximity extension assay	AUC (CLEC1B/CCL11) = 0.95, 95% CI = 0.86–1, p = 0.001<br>AUC (TGFa/CCL20) = 0.96, 95% CI = 0.88–1, p = 0.001	Diagnostic

Feinkohl et al., 2020 (60)	CC		N = 93<br>AD (44), CN (49)	Plasma	Aβ42/Aβ40	ELISA assays	AUC=0.59, Sn=61.2%, Sp=63.6%	Diagnostic
Gao et al., 2020 (61)	CC		N = 30<br>CN (10), MCI (10), AD (10)	Plasma, Serum	Aβ42/Aβ40	Nanosheet-based sensor system	*aMCI vs. Con:* AUC=0.83, Sn=70%, Sp=70%<br>*AD vs. Con:* AUC=0.91, Sn=90%, 90%<br>*aMCI vs. AD:*<br>AUC=0.75, Sn=80%, Sp=60%	Diagnostic
Janelidze et al., 2020 (62)	CC	BioFINDER, Arizona Study of Aging and Neurodegenerative Disorders/Brain and Body Donation Program	Cohort 1: 182<br>Cohort 2: 344<br>Cohort 3: 63	Plasma	Aβ42/Aβ40 + pTau181	MSD, Elecsys fully-automated immunoassays, Simoa	AUC=0.84 (95% CI = 0.79-0.89)	Diagnostic
Karikari et al., 2020 (63)	CC	TRIAD, BioFINDER-2, primary care cohort referred from primary care physicians of the Canadian National Health Service	N=1131<br>TRIAD cohort: 226<br>BioFINDER-2 cohort: 763<br>Primary care cohort: 105	Plasma	p-tau181	Ultrasensitive blood immunoassay	AUC = 85.3%; 95% CI 81.4–89.2%	Diagnostic, prognosis

		for specialist care.						
Palmqvist et al., 2020 (64)	CC	Arizona-based neuropathology cohort, Swedish BioFINDER-2 cohort, Colombian autosomal-dominant AD kindred	N = 1402	Plasma	p-tau217	Immunoassay	AUC = 0.89 and 0.96	Diagnostic
Perrotte et al., 2020 (65)	CC	Recruited from the Memory Clinic of Sherbrooke	N = 60	Plasma exosomes	p-tau181/t-tau ratio	Luminex assay	Control from AD [AUC of 0.823 ($p < 0.05$)<br>MCI from AD: AUC of 0.87 ($p < 0.05$)	Diagnostic, prognosis
Nam et al., 2020 (66)	Longitudinal study (4-year follow up)		N = 76<br>CN (26), MCI (30), AD (20)	Serum, Exosomes	T-tau, p-Tau202, p-tau202/T-tau	ELISA assays and Western blot	*Serum:*<br>AUC (T-tau) = 0.675<br>AUC (p-tau202) = 0.5958<br>AUC (p-tau202/T-tau) = 0.5253<br>*Exosomes:*<br>AUC (T-tau) = 0.6434<br>AUC (p-tau202) = 0.7333<br>AUC (p-tau202/T-tau) = 0.6113	Prognostic

Sheu et al., 2020 (67)	CC		N = 86<br>AD (32), CN (54)	Serum	AHI1	Western blot and ELISA assays	AUC=0.7-0.82	Diagnostic
Stockmann et al., 2020 (68)	Longitudinal study (6 years follow-up)	SCIENCe project and Amsterdam Dementia Cohort	N = 203	Plasma	Aβ misfolding + Aβ42/Aβ40[a]	Immuno-infrared-sensor technology and SIMOA	AUC (Aβ misfolding) =0.94 (95% CI, 0.86-1.00; 6-year follow-up)<br>AUC (Aβ42/Aβ40) = 0.92 (95% CI, 0.82-1.00)<br>AUC (panel) = 0.99 (95% CI, 0.99-1.00)	Prognostic
Teuber-Hanselmann et al., 2020 (69)	Multi-centre trans-sectional	Teuber-Hanselmann	N = 237<br>MCI (21), mild-AD (98), CN (118)	Serum	KLK8	ELISA assay	AUC(MCI)=0.94, Sn = 52.4%, Sp = 99.2%<br>AUC(AD)=0.83, Sn = 56.1%, Sp = 69.8%	Diagnostic
Verberk et al., 2020 (70)	CC		N = 252<br>SCD, MCI, AD	Plasma	Ab42/Ab40, GFAP, NF-L		Total (AUC): 0.88 (Sn = 0.82, Sp = 0.86)<br>Nondemented (AUC): 0.84 (Sn = 0.70, Sp = 0.86)	Prediction of amyloid PET status and disease monitoring
Wang et al., 2020 (71)	Pilot study, CC	Wang et al.	N = 30<br>MCI (10), AD (10), CN (10)	Plasma	AMPKα1	ELISA assay	AUC = 0.8 (MCI), 0.74 (AD), 0.77 (MCI + AD)<br>Sn = 88.89% (MCI), 77.78% (AD), 83.33% (MCI + AD)<br>Sp = 70% (MCI, AD)	Diagnostic
Westwood et al., 2020 (72)	CC	EMIF-AD catalogue	N = 1866<br>HC, MCI, AD	Plasma	FCN2, CFI, C4, B2M, Cathepsin D, APOE+4, A1AT	Luminex xMAP, ELISA, and Meso Scale Discovery assays	AUC = 0.742, Sn = 0.682, Sp = 0.704	Diagnostic
Youn et al., 2020 (73)	CC		N = 104<br>AD (52), CN (52)	Plasma	Aβ oligomers	Multimer detection system	Sn=100%, Sp=92.31%	Diagnostic
Jia et al., 2020 (74)	Two-stage-sectional study,		N = 739<br>Discovery stage: AD (28), aMCI (25),	Exosomes	GAP43 + Ng + SNAP-25	ELISA assays	AUC=0.87-0.89	Predictive

	longitudinal study	CN (29); Validation stage: AD (73), aMCI (71), CN (72)	
		preclinical AD (160), CN (160)	
		mutation carriers (59), non-mutation carriers (62)	

[a]Age, gender, and the presence of APOEε4 allele were included as covariates

References:

1. McGuinness B, Fuchs M, Barrett SL, Passmore AP, Johnston JA. Platelet Membrane β-Secretase Activity in Mild Cognitive Impairment and Conversion to Dementia: a Longitudinal Study. *J Alzheimers Dis* (2015) **49**:1095–1103. doi:10.3233/JAD-150795

2. Burnham SC, Rowe CC, Baker D, Bush AI, Doecke JD, Faux NG, Laws SM, Martins RN, Maruff P, Macaulay SL, et al. Predicting Alzheimer disease from a blood-based biomarker profile. *Neurology* (2016) **87**:1093–1101. doi:10.1212/WNL.0000000000003094

3. Janelidze S, Stomrud E, Palmqvist S, Zetterberg H, Van Westen D, Jeromin A, Song L, Hanlon D, Tan Hehir CA, Baker D, et al. Plasma β-amyloid in Alzheimer's disease and vascular disease. *Sci Rep* (2016) **6**: doi:10.1038/srep26801

4. Jammeh E, Zhao P, Carroll C, Pearson S, Ifeachor E. Identification of blood biomarkers for use in point of care diagnosis tool for Alzheimer's disease - IEEE Conference Publication. *2016 38th Annu Int Conf IEEE Eng Med Biol Soc* (2016) **2016**:2415–2418. doi:10.1109/EMBC.2016.7591217

5. Yu S, Liu YPY-HY-PH, Liu YPY-HY-PH, Jiao S-SS, Liu L, Wang Y-JJ, Fu W-LL. Diagnostic utility of VEGF and soluble CD40L levels in serum of Alzheimer's patients. *Clin Chim Acta* (2016) **453**:154–159. doi:10.1016/j.cca.2015.12.018

6. Zheng L, Kong X, Cui Y, Wei Y, Zhang J, Wei W. Conversion from MCI to AD in patients with the APOE ε4 genotype: Prediction by plasma HCY and serum BDNF. *Neurosci Lett* (2016) **626**:19–24. doi:10.1016/j.neulet.2016.05.018

7. An SSA, Lee BS, Yu JS, Lim K, Kim GJ, Lee R, Kim S, Kang S, Park YH, Wang MJ, et al. Dynamic changes of oligomeric amyloid β levels in plasma induced by spiked synthetic Aβ42. *Alzheimer's Res Ther* (2017) **9**:1–10. doi:10.1186/S13195-017-0310-6/FIGURES/4

8. Goudey B, Fung BJ, Schieber C, Faux NG. A blood-based signature of cerebrospinal fluid Aβ1−42 status. *bioRxiv* (2017) doi:10.1101/190207

9. Janel N, Alexopoulos P, Badel A, Lamari F, Camproux AC, Lagarde J, Simon S, Feraudet-Tarisse C, Lamourette P, Arbones M, et al. Combined assessment of DYRK1A, BDNF and homocysteine levels as diagnostic marker for Alzheimer's disease. *Transl Psychiatry* (2017) **7**:e1154–e1154. doi:10.1038/tp.2017.123

10. Mattsson N, Andreasson U, Zetterberg H, Blennow K, Weiner MW, Aisen P, Toga AW, Petersen R, Jack CR, Jagust W, et al. Association of plasma neurofilament light with neurodegeneration in patients with Alzheimer disease. *JAMA Neurol* (2017) **74**:557–566. doi:10.1001/jamaneurol.2016.6117

11. Mohd Hasni DS, Lim SM, Chin AV, Tan MP, Poi PJH, Kamaruzzaman SB, Majeed ABA, Ramasamy K. Peripheral cytokines, C-X-C motif ligand10 and interleukin-13, are associated with Malaysian Alzheimer's disease. *Geriatr Gerontol Int* (2017)

17:839–846. doi:10.1111/ggi.12783

12. Pedrini S, Gupta VB, Hone E, Doecke J, O'Bryant S, James I, Bush AI, Rowe CC, Villemagne VL, Ames D, et al. A blood-based biomarker panel indicates IL-10 and IL-12/23p40 are jointly associated as predictors of β-amyloid load in an AD cohort. *Sci Rep* (2017) **7**:14057. doi:10.1038/s41598-017-14020-9

13. Popp J, Oikonomidi A, Tautvydaitė D, Dayon L, Bacher M, Migliavacca E, Henry H, Kirkland R, Severin I, Wojcik J, et al. Markers of neuroinflammation associated with Alzheimer's disease pathology in older adults. *Brain Behav Immun* (2017) **62**:203–211. doi:10.1016/j.bbi.2017.01.020

14. Tateno A, Sakayori T, Kim WC, Koeda M, Kumita S, Suzuki H, Okubo Y. Effect of apolipoprotein E phenotype on the association of plasma amyloid β and amyloid positron emission tomography imaging in Japan. *Alzheimer's Dement Diagnosis, Assess Dis Monit* (2017) **9**:51–56. doi:10.1016/j.dadm.2017.08.002

15. Wang MJ, Yi S, Han J-Y, Park SY, Jang J-W, Chun IK, Kim SE, Lee BS, Kim GJ, Yu JS, et al. Oligomeric forms of amyloid-β protein in plasma as a potential blood-based biomarker for Alzheimer's disease. *Alzheimers Res Ther* (2017) **9**:98. doi:10.1186/s13195-017-0324-0

16. Yu S, Liu Y-P, Liu H-L, Li J, Xiang Y, Liu Y-H, Jiao S-S, Liu L, Wang Y, Fu W. Serum Protein-Based Profiles as Novel Biomarkers for the Diagnosis of Alzheimer's Disease. *Mol Neurobiol* (2017) **55**:3999–4008. doi:10.1007/s12035-017-0609-0

17. Chen K, Gao T, Bai Z, Yuan Z. Circulating APP, NCAM and Aβ serve as biomarkers for Alzheimer's disease. *Brain Res* (2018) **1699**:117–120. doi:10.1016/j.brainres.2018.08.015

18. Cheng Z, Yin J, Yuan H, Jin C, Zhang F, Wang Z, Liu X, Wu Y, Wang T, Xiao S. Blood-Derived Plasma Protein Biomarkers for Alzheimer's Disease in Han Chinese. *Front Aging Neurosci* (2018) **10**:414. doi:10.3389/fnagi.2018.00414

19. de Rojas I, Romero J, Rodríguez-Gomez O, Pesini P, Sanabria A, Pérez-Cordon A, Abdelnour C, Hernández I, Rosende-Roca M, Mauleón A, et al. Correlations between plasma and PET beta-amyloid levels in individuals with subjective cognitive decline: the Fundació ACE Healthy Brain Initiative (FACEHBI). *Alzheimers Res Ther* (2018) **10**:119. doi:10.1186/s13195-018-0444-1

20. Eke CS, Jammeh E, Li X, Carroll C, Pearson S, Ifeachor E. Identification of Optimum Panel of Blood-based Biomarkers for Alzheimer's Disease Diagnosis Using Machine Learning. in *2018 40th Annual International Conference of the IEEE Engineering in Medicine and Biology Society (EMBC)* (United States: IEEE), 3991–3994. doi:10.1109/EMBC.2018.8513293

21. Lewczuk P, Ermann N, Andreasson U, Schultheis C, Podhorna J, Spitzer P, Maler JM, Kornhuber J, Blennow K, Zetterberg H. Plasma neurofilament light as a potential biomarker of neurodegeneration in Alzheimer's disease. *Alzheimers Res Ther* (2018) **10**:71. doi:10.1186/s13195-018-0404-9

22. Mielke MM, Hagen CE, Xu J, Chai X, Vemuri P, Lowe VJ, Airey DC, Knopman DS, Roberts RO, Machulda MM, et al. Plasma phospho-tau181 increases with Alzheimer's disease clinical severity and is associated with tau- and amyloid-positron emission

tomography. *Alzheimer's Dement* (2018) **14**:989–997. doi:10.1016/j.jalz.2018.02.013

23. Nabers A, Perna L, Lange J, Mons U, Schartner J, Güldenhaupt J, Saum K-U, Janelidze S, Holleczek B, Rujescu D, et al. Amyloid blood biomarker detects Alzheimer's disease. *EMBO Mol Med* (2018) **10**: doi:10.15252/emmm.201708763

24. Nakamura A, Kaneko N, Villemagne VL, Kato T, Doecke J, Doré V, Fowler C, Li QX, Martins R, Rowe C, et al. High performance plasma amyloid-β biomarkers for Alzheimer's disease. *Nature* (2018) **554**:249–254. doi:10.1038/nature25456

25. Shen Y, Wang H, Sun Q, Yao H, Keegan AP, Mullan M, Wilson J, Lista S, Leyhe T, Laske C, et al. Increased Plasma Beta-Secretase 1 May Predict Conversion to Alzheimer's Disease Dementia in Individuals With Mild Cognitive Impairment. *Biol Psychiatry* (2018) **83**:447–455. doi:10.1016/j.biopsych.2017.02.007

26. Verberk IMW, Slot RE, Verfaillie SCJ, Heijst H, Prins ND, van Berckel BNM, Scheltens P, Teunissen CE, van der Flier WM. Plasma Amyloid as Prescreener for the Earliest <scp>A</scp> lzheimer Pathological Changes. *Ann Neurol* (2018) **84**:648–658. doi:10.1002/ana.25334

27. Winston CN, Goetzl EJ, Baker LD, Vitiello M V., Rissman RA. Growth Hormone-Releasing Hormone Modulation of Neuronal Exosome Biomarkers in Mild Cognitive Impairment. *J Alzheimer's Dis* (2018) **66**:971–981. doi:10.3233/JAD-180302

28. Agliardi C, Guerini FR, Zanzottera M, Bianchi A, Nemni R, Clerici M. SNAP-25 in Serum Is Carried by Exosomes of Neuronal Origin and Is a Potential Biomarker of Alzheimer's Disease. *Mol Neurobiol* (2019) **56**:5792–5798. doi:10.1007/s12035-019-1501-x

29. Baldacci F, Daniele S, Piccarducci R, Giampietri L, Pietrobono D, Giorgi FS, Nicoletti V, Frosini D, Libertini P, Lo Gerfo A, et al. Potential Diagnostic Value of Red Blood Cells α-Synuclein Heteroaggregates in Alzheimer's Disease. *Mol Neurobiol* (2019) **56**:6451–6459. doi:10.1007/s12035-019-1531-4

30. Boccardi V, Paolacci L, Remondini D, Giampieri E, Poli G, Curti N, Cecchetti R, Villa A, Ruggiero C, Brancorsini S, et al. Cognitive Decline and Alzheimer's Disease in Old Age: A Sex-Specific Cytokinome Signature. *J Alzheimer's Dis* (2019) **72**:911–918. doi:10.3233/JAD-190480

31. Bram JM de F, Talib LL, Joaquim HPG, Sarno TA, Gattaz WF, Forlenza OV. Protein levels of ADAM10, BACE1, and PSEN1 in platelets and leukocytes of Alzheimer's disease patients. *Eur Arch Psychiatry Clin Neurosci* (2019) **269**:963–972. doi:10.1007/s00406-018-0905-3

32. Chatterjee P, Elmi M, Goozee K, Shah T, Sohrabi HR, Dias CB, Pedrini S, Shen K, Asih PR, Dave P, et al. Ultrasensitive Detection of Plasma Amyloid-β as a Biomarker for Cognitively Normal Elderly Individuals at Risk of Alzheimer's Disease. *J Alzheimer's Dis* (2019) **71**:775–783. doi:10.3233/JAD-190533

33. Chen Z, Mengel D, Keshavan A, Rissman RA, Billinton A, Perkinton M, Percival-Alwyn J, Schultz A, Properzi M, Johnson K, et al. Learnings about the complexity of extracellular tau aid development of a blood-based screen for Alzheimer's disease.

Alzheimer's Dement (2019) **15**:487–496. doi:10.1016/j.jalz.2018.09.010

34. Guzmán-Martínez L, Tapia JP, Farías GA, González A, Estrella M, MacCioni RB. The Alz-tau Biomarker for Alzheimer's Disease: Study in a Caucasian Population. *J Alzheimer's Dis* (2019) **67**:1181–1186. doi:10.3233/JAD-180637

35. Han S-HH, Park J-CC, Byun MS, Yi D, Lee JH, Lee DY, Mook-Jung I. Blood acetylcholinesterase level is a potential biomarker for the early detection of cerebral amyloid deposition in cognitively normal individuals. *Neurobiol Aging* (2019) **73**:21–29. doi:10.1016/j.neurobiolaging.2018.09.001

36. Iulita MF, Ganesh A, Pentz R, Flores Aguilar L, Gubert P, Ducatenzeiler A, Christie S, Wilcock GK, Cuello AC. Identification and Preliminary Validation of a Plasma Profile Associated with Cognitive Decline in Dementia and At-Risk Individuals: A Retrospective Cohort Analysis. *J Alzheimer's Dis* (2019) **67**:327–341. doi:10.3233/JAD-180970

37. Jia L, Qiu Q, Zhang H, Chu L, Du Y, Zhang J, Zhou C, Liang F, Shi S, Wang S, et al. Concordance between the assessment of Aβ42, T-tau, and P-T181-tau in peripheral blood neuronal-derived exosomes and cerebrospinal fluid. *Alzheimers Dement* (2019) **15**:1071–1080. doi:10.1016/j.jalz.2019.05.002

38. Kapogiannis D, Mustapic M, Shardell MD, Berkowitz ST, Diehl TC, Spangler RD, Tran J, Lazaropoulos MP, Chawla S, Gulyani S, et al. Association of Extracellular Vesicle Biomarkers with Alzheimer Disease in the Baltimore Longitudinal Study of Aging. *JAMA Neurol* (2019) **76**:1340–1351. doi:10.1001/jamaneurol.2019.2462

39. Li W-WW, Shen Y-YY, Tian D-YY, Bu X-L Le, Zeng F, Liu Y-HH, Chen Y, Yao X-QQ, Li H-YY, Chen D-WW, et al. Brain Amyloid-β deposition and blood biomarkers in patients with clinically diagnosed alzheimer's disease. *J Alzheimer's Dis* (2019) **69**:169–178. doi:10.3233/JAD-190056

40. Liu S, Suzuki H, Ito H, Korenaga T, Akatsu H, Meno K, Uchida K. Serum levels of proteins involved in amyloid-β clearance are related to cognitive decline and neuroimaging changes in mild cognitive impairment. *Alzheimer's Dement Diagnosis, Assess Dis Monit* (2019) **11**:85–97. doi:10.1016/j.dadm.2018.11.003

41. Liu S, Pan J, Tang K, Lei Q, He L, Cai X, Li Z. Alpha 1-antichymotrypsin may be a biomarker for the progression of amnestic mild cognitive impairment. *Acta Neurol Belg* (2019) doi:10.1007/s13760-019-01206-3

42. Meng X, Li T, Wang X, Lv X, Sun Z, Zhang J, Su F, Kang S, Kim S, An SSA, et al. Association between increased levels of amyloid-β oligomers in plasma and episodic memory loss in Alzheimer's disease. *Alzheimers Res Ther* (2019) **11**:89. doi:10.1186/s13195-019-0535-7

43. Morgan AR, Touchard S, Leckey C, O'Hagan C, Nevado-Holgado AJ, Barkhof F, Bertram L, Blin O, Bos I, Dobricic V, et al. Inflammatory biomarkers in Alzheimer's disease plasma. *Alzheimer's Dement* (2019) **15**:776–787. doi:10.1016/j.jalz.2019.03.007

44. Nabers A, Hafermann H, Wiltfang J, Gerwert K. Aβ and tau structure-based biomarkers for a blood- and CSF-based two-step

recruitment strategy to identify patients with dementia due to Alzheimer's disease. *Alzheimer's Dement Diagnosis, Assess Dis Monit* (2019) **11**:257–263. doi:10.1016/j.dadm.2019.01.008

45. Palmqvist S, Janelidze S, Stomrud E, Zetterberg H, Karl J, Zink K, Bittner T, Mattsson N, Eichenlaub U, Blennow K, et al. Performance of Fully Automated Plasma Assays as Screening Tests for Alzheimer Disease–Related β-Amyloid Status. *JAMA Neurol* (2019) **76**:1060. doi:10.1001/jamaneurol.2019.1632

46. Park J-C, Han S-H, Yi D, Byun MS, Lee JH, Jang S, Ko K, Jeon SY, Lee Y-S, Kim YK, et al. Plasma tau/amyloid-β1–42 ratio predicts brain tau deposition and neurodegeneration in Alzheimer's disease. *Brain* (2019) **142**:771–786. doi:10.1093/brain/awy347

47. Park JC, Han SH, Lee H, Jeong H, Byun MS, Bae J, Kim H, Lee DY, Yi D, Shin SA, et al. Prognostic plasma protein panel for Aβ deposition in the brain in Alzheimer's disease. *Prog Neurobiol* (2019) **183**:101690. doi:10.1016/j.pneurobio.2019.101690

48. Pérez-Grijalba V, Arbizu J, Romero J, Prieto E, Pesini P, Sarasa L, Guillen F, Monleón I, San-José I, Martínez-Lage P, et al. Plasma Aβ42/40 ratio alone or combined with FDG-PET can accurately predict amyloid-PET positivity: a cross-sectional analysis from the AB255 Study. *Alzheimers Res Ther* (2019) **11**:96. doi:10.1186/s13195-019-0549-1

49. Perrotte M, Le Page A, Fournet M, Le Sayec M, Rassart É, Fulop T, Ramassamy C. Blood-based redox-signature and their association to the cognitive scores in MCI and Alzheimer's disease patients. *Free Radic Biol Med* (2019) **130**:499–511. doi:10.1016/j.freeradbiomed.2018.10.452

50. Schindler SE, Bollinger JG, Ovod V, Mawuenyega KG, Li Y, Gordon BA, Holtzman DM, Morris JC, Benzinger TLS, Xiong C, et al. High-precision plasma β-amyloid 42/40 predicts current and future brain amyloidosis. *Neurology* (2019) **93**:E1647–E1659. doi:10.1212/WNL.0000000000008081

51. Schipke CG, Günter O, Weinert C, Scotton P, Sigle J-P, Kallarackal J, Kabelitz D, Finzen A, Feuerhelm-Heidl A. Definition and quantification of six immune- and neuroregulatory serum proteins in healthy and demented elderly. *Neurodegener Dis Manag* (2019) **9**:193–203. doi:10.2217/nmt-2019-0003

52. Whelan CD, Mattsson N, Nagle MW, Vijayaraghavan S, Hyde C, Janelidze S, Stomrud E, Lee J, Fitz L, Samad TA, et al. Multiplex proteomics identifies novel CSF and plasma biomarkers of early Alzheimer's disease. *Acta Neuropathol Commun* (2019) **7**:169. doi:10.1186/s40478-019-0795-2

53. Yao F, Zhang K, Zhang Y, Guo Y, Li A, Xiao S, Liu Q, Shen L, Ni J. Identification of blood biomarkers for Alzheimer's disease through computational prediction and experimental validation. *Front Neurol* (2019) **10**:1158. doi:10.3389/fneur.2018.01158

54. Abate G, Vezzoli M, Polito L, Guaita A, Albani D, Marizzoni M, Garrafa E, Marengoni A, Forloni G, Frisoni GB, et al. A conformation variant of p53 combined with machine learning identifies alzheimer disease in preclinical and prodromal stages. *J Pers Med* (2021) **11**:1–16. doi:10.3390/jpm11010014

55. Abe K, Shang J, Shi X, Yamashita T, Hishikawa N, Takemoto M, Morihara R, Nakano Y, Ohta Y, Deguchi K, et al. A New Serum Biomarker Set to Detect Mild Cognitive Impairment and Alzheimer's Disease by Peptidome Technology. *J Alzheimer's Dis* (2020) **73**:217–227. doi:10.3233/JAD-191016

56. Barthélemy NR, Horie K, Sato C, Bateman RJ. Blood plasma phosphorylated-tau isoforms track CNS change in Alzheimer's disease. *J Exp Med* (2020) **217**: doi:10.1084/jem.20200861

57. Doecke JD, Pérez-Grijalba V, Fandos N, Fowler C, Villemagne VL, Masters CL, Pesini P, Sarasa M. Total Aβ42/Aβ40 ratio in plasma predicts amyloid-PET status, independent of clinical AD diagnosis. *Neurology* (2020) **94**:E1580–E1591. doi:10.1212/WNL.0000000000009240

58. Eke CS, Sakr F, Jammeh E, Zhao P, Ifeachor E. A Robust Blood-based Signature of Cerebrospinal Fluid Aβ(42) Status. *Annu Int Conf IEEE Eng Med Biol Soc IEEE Eng Med Biol Soc Annu Int Conf* (2020) **2020**:5523–5526. doi:10.1109/EMBC44109.2020.9175158

59. Ellegaard Nielsen J, Sofie Pedersen K, Vestergård K, Georgiana Maltesen R, Christiansen G, Lundbye-Christensen S, Moos T, Risom Kristensen S, Pedersen S. Novel Blood-Derived Extracellular Vesicle-Based Biomarkers in Alzheimer's Disease Identified by Proximity Extension Assay. *Biomedicines* (2020) **8**:199. doi:10.3390/biomedicines8070199

60. Feinkohl I, Schipke CG, Kruppa J, Menne F, Winterer G, Pischon T, Peters O. Plasma Amyloid Concentration in Alzheimer's Disease: Performance of a High-Throughput Amyloid Assay in Distinguishing Alzheimer's Disease Cases from Controls. *J Alzheimer's Dis* (2020) **74**:1285–1294. doi:10.3233/JAD-200046

61. Gao H, Liu M, Zhao Z, Yang C, Zhu L, Cai Y, Yang Y, Hu Z. Diagnosis of Mild Cognitive Impairment and Alzheimer's Disease by the Plasma and Serum Amyloid-beta 42 Assay through Highly Sensitive Peptoid Nanosheet Sensor. *ACS Appl Mater Interfaces* (2020) **12**:9693–9700. doi:10.1021/acsami.0c00370

62. Janelidze S, Mattsson N, Palmqvist S, Smith R, Beach TG, Serrano GE, Chai X, Proctor NK, Eichenlaub U, Zetterberg H, et al. Plasma P-tau181 in Alzheimer's disease: relationship to other biomarkers, differential diagnosis, neuropathology and longitudinal progression to Alzheimer's dementia. *Nat Med* (2020) **26**:379–386. doi:10.1038/s41591-020-0755-1

63. Karikari TK, Pascoal TA, Ashton NJ, Janelidze S, Benedet AL, Rodriguez JL, Chamoun M, Savard M, Kang MS, Therriault J, et al. Blood phosphorylated tau 181 as a biomarker for Alzheimer's disease: a diagnostic performance and prediction modelling study using data from four prospective cohorts. *Lancet Neurol* (2020) **19**:422–433. doi:10.1016/S1474-4422(20)30071-5

64. Palmqvist S, Janelidze S, Quiroz YT, Zetterberg H, Lopera F, Stomrud E, Su Y, Chen Y, Serrano GE, Leuzy A, et al. Discriminative Accuracy of Plasma Phospho-tau217 for Alzheimer Disease vs Other Neurodegenerative Disorders. *JAMA* (2020) **324**:772–781. doi:10.1001/JAMA.2020.12134

65. Perrotte M, Haddad M, Le Page A, Frost EH, Fulöp T, Ramassamy C. Profile of pathogenic proteins in total circulating extracellular vesicles in mild cognitive impairment and during the progression of Alzheimer's disease. *Neurobiol Aging* (2020) **86**:102–111. doi:10.1016/j.neurobiolaging.2019.10.010

66. Nam E, Lee Y-B, Moon C, Chang K-A. Serum Tau Proteins as Potential Biomarkers for the Assessment of Alzheimer's Disease Progression. *Int J Mol Sci* (2020) **21**:5007. doi:10.3390/ijms21145007

67. Sheu JJ, Yang LY, Sanotra MR, Wang S Te, Lu HT, Kam RSY, Hsu IU, Kao SH, Lee CK, Shieh JCC, et al. Reduction of AHI1 in the serum of Taiwanese with probable Alzheimer's disease. *Clin Biochem* (2020) **76**:24–30. doi:10.1016/j.clinbiochem.2019.11.011

68. Stockmann J, Verberk IMW, Timmesfeld N, Denz R, Budde B, Lange-Leifhelm J, Scheltens P, van der Flier WM, Nabers A, Teunissen CE, et al. Amyloid-β misfolding as a plasma biomarker indicates risk for future clinical Alzheimer's disease in individuals with subjective cognitive decline. *Alzheimers Res Ther* (2020) **12**:169. doi:10.1186/s13195-020-00738-8

69. Teuber-Hanselmann S, Rekowski J, Vogelgsang J, Von Arnim C, Reetz K, Stang A, Jöckel KH, Wiltfang J, Esselmann H, Otto M, et al. CSF and blood kallikrein-8: A promising early biomarker for Alzheimer's disease. *J Neurol Neurosurg Psychiatry* (2020) **91**:40–48. doi:10.1136/jnnp-2019-321073

70. Verberk IMW, Thijssen E, Koelewijn J, Mauroo K, Vanbrabant J, de Wilde A, Zwan MD, Verfaillie SCJ, Ossenkoppele R, Barkhof F, et al. Combination of plasma amyloid beta(1-42/1-40) and glial fibrillary acidic protein strongly associates with cerebral amyloid pathology. *Alzheimers Res Ther* (2020) **12**:118. doi:10.1186/s13195-020-00682-7

71. Wang X, Zimmermann HR, Lockhart SN, Craft S, Ma T. Decreased Levels of Blood AMPKα1 but not AMPKα2 Isoform in Patients with Mild Cognitive Impairment and Alzheimer's Disease: A Pilot Study. *J Alzheimer's Dis* (2020) **76**:217–224. doi:10.3233/JAD-191189

72. Westwood S, Baird AL, Anand SN, Nevado-Holgado AJ, Kormilitzin A, Shi L, Hye A, Ashton NJ, Morgan AR, Bos I, et al. Validation of Plasma Proteomic Biomarkers Relating to Brain Amyloid Burden in the EMIF-Alzheimer's Disease Multimodal Biomarker Discovery Cohort. *J Alzheimer's Dis* (2020) **74**:213–225. doi:10.3233/JAD-190434

73. Youn YC, Lee BS, Kim GJ, Ryu JS, Lim K, Lee R, Suh J, Park YH, Pyun J-M, Ryu N, et al. Blood Amyloid-β Oligomerization as a Biomarker of Alzheimer's Disease: A Blinded Validation Study. *J Alzheimer's Dis* (2020) **75**:493–499. doi:10.3233/jad-200061

74. Jia L, Zhu M, Kong C, Pang Y, Zhang H, Qiu Q, Wei C, Tang Y, Wang Q, Li Y, et al. Blood neuro-exosomal synaptic proteins predict Alzheimer's disease at the asymptomatic stage. *Alzheimer's Dement* (2021) **17**:49–60. doi:10.1002/alz.12166
