# Supplementary material for: Diagnostic Accuracy of Blood-Based Biomarker Panels: A Systematic Review
Source: Front Aging Neurosci. 2022 Mar 11;14:683689. doi: 10.3389/fnagi.2022.683689 (PMC8963375; doi:10.3389/fnagi.2022.683689)

Supplementary Material

Supplementary Table 2. Performance of blood biomarker panels for diagnosing AD

Biomarker panel	Reference	N total	N disease	N control	AUC	AUC weighted mean	SE (AUC)	Q1	Q2			
Aβ42/Aβ40, APOEε4 status	Doecke et al., 2020	176	46	130	0,896		0,032	0,812	0,847	6,169	5980,000	0,001
		169	46	123	0,88		0,034	0,786	0,824	6,645	5658,000	0,001
		135	31	104	0,913		0,036	0,840	0,871	4,175	3224,000	0,001
	Schindler et al., 2019	158	43	115	0,94		0,026	0,887	0,911	3,306	4945,000	0,001
	Verberk et al., 2018	248	57	191	0,79		0,038	0,653	0,697	15,690	10887,000	0,001
		248	57	191	0,83		0,035	0,709	0,753	13,448	10887,000	0,001
	Chatterjee et al., 2019	95	72	23	0,78		0,049	0,639	0,684	4,023	1656,000	0,002
	Tateno et al., 2017	117	93	24	0,519		0,066	0,350	0,355	9,671	2232,000	0,004
		117	93	24	0,648		0,059	0,479	0,510	7,754	2232,000	0,003
						0,814	0,040					
Aβ42/Aβ40, ptau 181	Janelidze et al., 2020	182	104	78	0,84	0,84	0,029	0,724	0,767	6,768	8112,000	0,001
Aβ42, CgA, EOT3, APOEε4 status	Eke et al., 2020	358	300	58	0,84	0,84	0,023	0,724	0,767	9,175	17400,000	0,001
Aβ42, APOE, CgA, EOT3, APOEε4 status	Goudey et al., 2017	358	300	58	0,84	0,84	0,023	0,724	0,767	9,175	17400,000	0,001
Brain derived exosome Aβ42, pTau181, T-tau	Jia et al., 2019	216	144	72	0,85		0,025	0,739	0,781	6,665	10368,000	0,001
		216	144	72	0,98		0,009	0,961	0,970	0,763	10368,000	0,000
		216	144	72	0,98		0,009	0,961	0,970	0,763	10368,000	0,000
		216	144	72	0,86	0,918	0,024	0,754	0,795	6,187	10368,000	0,001

$$SE(AUC) = \sqrt{\frac{AUC(1-AUC) + (N_1 - 1)(Q_1 - AUC^2) + (N_2 - 1)(Q_2 - AUC^2)}{N_1 N_2}}$$

$$Q_1 = \frac{AUC}{2 - AUC}$$

$$Q_2 = \frac{2AUC^2}{1 + AUC}$$

Hanley and McNeil (1982) proposed formula to calculate AUC standard error (SE)

Biomarker panel	AUC weighted mean	SE (AUC)
Aβ42/Aβ40, APOEε4 status	81,4%	0,040
Aβ42/Aβ40, pTau 181	84,0%	0,029
Aβ42, CgA, EOT3, APOEε4 status	84,0%	0,023
Aβ42, APOE, CgA, EOT3, APOEε4 status	84,0%	0,023
Brain derived exosomal Aβ42, pTau181, T-tau	91,8%	0,024

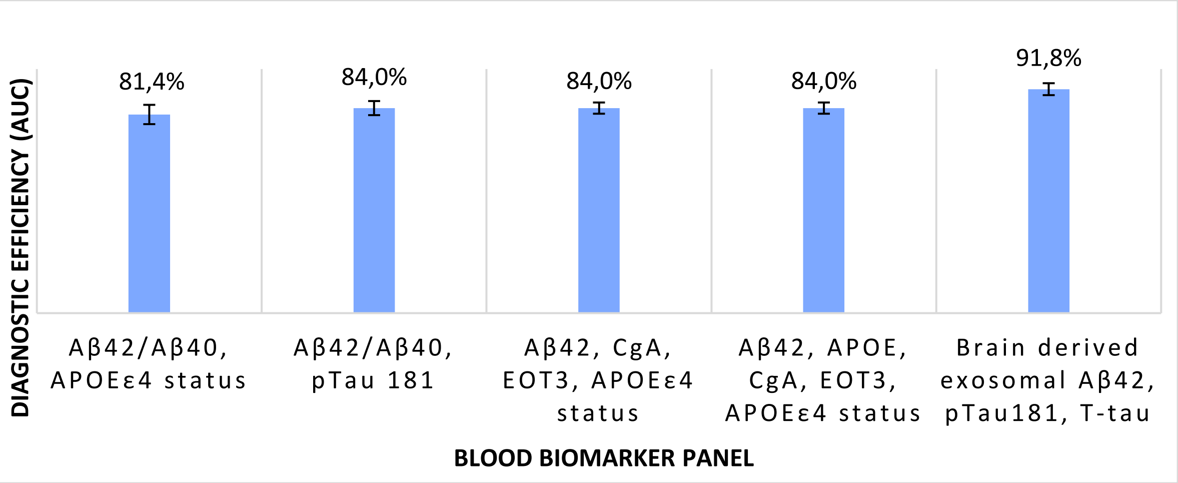

Supplement: Supplementary file 2 [file Data_Sheet_1.PDF]
